# Genetic variation of six desaturase genes in flax and their impact on fatty acid composition

**DOI:** 10.1007/s00122-013-2161-2

**Published:** 2013-08-09

**Authors:** Dinushika Thambugala, Scott Duguid, Evelyn Loewen, Gordon Rowland, Helen Booker, Frank M. You, Sylvie Cloutier

**Affiliations:** 1 Department of Plant Science, University of Manitoba, 66 Dafoe Rd, Winnipeg, MB R3T 2N2 Canada; 2 Morden Research Station, Agriculture and Agri-Food Canada, 101 Route 100, Unit 100, Morden, MB R6M 1Y5 Canada; 3 Crop Development Centre, University of Saskatchewan, 51 Campus Drive, Saskatoon, SK S7N 5A8 Canada; 4 Cereal Research Centre, Agriculture and Agri-Food Canada, 195 Dafoe Rd, Winnipeg, MB R3T 2M9 Canada

## Abstract

Flax (*Linum usitatissimum* L.) is one of the richest plant sources of omega-3 fatty acids praised for their health benefits. In this study, the extent of the genetic variability of genes encoding stearoyl-ACP desaturase (SAD), and fatty acid desaturase 2 (FAD2) and 3 (FAD3) was determined by sequencing the six paralogous genes from 120 flax accessions representing a broad range of germplasm including some EMS mutant lines. A total of 6 alleles for *sad1* and *sad2*, 21 for *fad2a*, 5 for *fad2b*, 15 for *fad3a* and 18 for *fad3b* were identified. Deduced amino acid sequences of the alleles predicted 4, 2, 3, 4, 6 and 7 isoforms, respectively. Allele frequencies varied greatly across genes. *Fad3a*, with 110 SNPs and 19 indels, and *fad3b*, with 50 SNPs and 5 indels, showed the highest levels of genetic variations. While most of the SNPs and all the indels were silent mutations, both genes carried nonsense SNP mutations resulting in premature stop codons, a feature not observed in *sad* and *fad2* genes. Some alleles and isoforms discovered in induced mutant lines were absent in the natural germplasm. Correlation of these genotypic data with fatty acid composition data of 120 flax accessions phenotyped in six field experiments revealed statistically significant effects of some of the SAD and FAD isoforms on fatty acid composition, oil content and iodine value. The novel allelic variants and isoforms identified for the six desaturases will be a resource for the development of oilseed flax with unique and useful fatty acid profiles.

**Electronic supplementary material:**

The online version of this article (doi:10.1007/s00122-013-2161-2) contains supplementary material, which is available to authorized users.

## Introduction

Flax (*Linum usitatissimum* L.) is an annual, self-pollinating, diploid (2*n* = 2*x* = 30) crop belonging to the Linaceae family. Flax has been grown for its stem fibers (fiber flax) or its seed oil (linseed or oilseed flax) for several thousand years (Zohary 1999). During the last two decades, flax has attracted great attention to human health mostly because of its desirable fatty acid composition. Current linseed varieties have oil content up to 50 % (Cloutier et al. 2010) and the major fatty acids are palmitic (PAL, C16:0; ~6 %), stearic (STE, C18:0; ~4.4 %), oleic (OLE, C18:1cis^∆9^; ~24.2 %), linoleic (LIO, C18:2cis^∆9,12^; ~15.3 %) and linolenic (LIN, C18:3 cis^∆9,12,15^; ~50.1 %) (Muir and Westcott 2003). Flax is the leading source of plant-based omega-3 fatty acids. Alpha-linolenic acid (ALA), the parent fatty acid of the omega-3 family, constitutes up to 73 % of the total fatty acids in high-LIN varieties, whereas traditional linseed varieties have 50–59 % ALA. Solin-type flax varieties are rich in LIO, the parent fatty acid of the omega-6 family, and contain generally 2–4 % ALA (Fofana et al. 2010).

LIO and LIN, important plant polyunsaturated fatty acids (PUFAs), are involved in plant metabolism as structural components, source of energy storage in the form of triacylglycerols (TAGs), essential components of cell membranes and precursors of signaling molecules such as jasmonic acid (Ohlrogge and Browse 1995). Mammalian tissues cannot synthesize LIO and LIN and hence these fatty acids are considered essential. Upon ingestion, LIO and LIN can be further elongated and desaturated to form other long chain PUFAs (LCPUFAs) such as eicosapentaenoic acid (EPA, C22:5), docosahexaenoic acid (DHA, C22:6) and arachidonic acid (AA, C20:4) (Warude et al. 2006). These LCPUFAs are essential structural components of biological membranes, especially in the brain and the retina, and are associated with developmental and physiological processes that affect human health (Dyer et al. 2008). DHA plays a vital role in brain development in infants and in normal brain function in adults (Martinetz 1992). LCPUFAs are important in maintaining the flexibility, fluidity and selective permeability of cellular membranes and their roles in preventing cardiovascular diseases and reducing bad cholesterol levels have been shown (Ander et al. 2004; Wiesenfeld et al. 2003). The insufficient amount of ALA in the typical Western diets is a major concern in cardiovascular diseases (Simopoulos 2000; Lands 2001).

Fatty acid desaturases and elongases are key enzymes involved in the fatty acid biosynthesis pathway (Warude et al. 2006). During oil biosynthesis in plants, the stepwise desaturation of fatty acids is an important process that determines the saturated to unsaturated fatty acid ratio and, ultimately, the end use of the oil as a food source or for industrial applications (Knutzon et al. 1992; Mikkilineni and Rocheford 2003). Fatty acid desaturases are responsible for the insertion of double bonds into the hydrocarbon chain of fatty acids (Shanklin and Cahoon 1998; Los and Murata 1998). Fatty acid desaturases, FAD2 and FAD3, are membrane-bound proteins with three highly conserved histidine box motifs essential for enzyme activity (Shanklin et al. 1994; Los and Murata 1998), while stearoyl-ACP desaturase (SAD) is the only known soluble desaturase with two characteristic HIS-box motifs (Singh et al. 1994; Luo et al. 2009; Shilman et al. 2011). Genes encoding desaturases involved in the fatty acid biosynthesis pathway have been cloned and characterized from many species (Chi et al. 2008; Lu et al. 2010; Chen et al. 2010).

Many of the genes encoding the enzymes that perform de novo fatty acid biosynthesis in flax have also been identified and characterized (Green 1986a; Fofana et al. 2004; Sorensen et al. 2005; Vrinten et al. 2005; Fofana et al. 2006; Krasowska et al. 2007; Khadake et al. 2009; Banik et al. 2011). SAD is responsible for converting stearoyl-ACP to oleoyl-ACP by introducing a double bond at the ∆9 position and thereby has the potential to increase the unsaturated FA content of the plant (Ohlrogge and Jaworski 1997). Two paralogous *sad* loci, *sad1* and *sad2*, differentially expressed in plants, have been identified in flax (Jain et al. 1999). Singh et al. (1994) reported the isolation and characterization of a cDNA sequence encoding the SAD protein from flax cultivar Glenelg, and Fofana et al. (2004) from AC McDuff. The *fad2* genes encode proteins responsible for desaturation of OLE into LIO by addition of a double bond at the ∆12 position. Two closely related *fad2* genes, namely, *fad2a* and *fad2b*, were cloned and characterized from flax genotypes Nike and NL97 (Krasowska et al. 2007; Khadake et al. 2009). The *fad3* genes encode proteins responsible for the desaturation of LIO into LIN by performing the addition of a double bond at the ∆15 position. Three *fad3* genes have been identified in the flax genome: *fad3a* and *fad3b* from cultivar Normandy (Vrinten et al. 2005) and more recently *fad3c* (Banik et al. 2011). FAD3A and FAD3B have been shown to be the major enzymes controlling the LIN content of the storage lipids in flaxseeds (Vrinten et al. 2005), while a major role for FAD3C has not been established.

While our knowledge of the major desaturase genes and enzymes involved in the fatty acid composition of flaxseeds is good, little is known about the extent of the genetic variability of these genes, their corresponding isoforms and their relationship to fatty acid composition. The major aim of this study was to determine the genetic variation for *sad*, *fad2* and *fad3* genes in flax by sequencing these genes from 120 flax accessions. These genotypic data were correlated to the fatty acid composition phenotyped in multiple field experiments during three years at two locations to hypothesize the functionality of these alleles and isoforms.

## Materials and methods

### Plant material and DNA extraction

A total of 120 *Linum usitatissimum* (L.) accessions representing both oil and fiber types of flax were selected for this study (ESM 1). Seeds obtained from the Plant Gene Resources of Canada (PGRC) were grown in a greenhouse. DNA was extracted from lyophilized young leaf tissues using the DNeasy 96 Plant kit (Qiagen, Mississauga, ON, Canada) according to the manufacturer’s instructions and quantified by fluorometry.

### Primer design and PCR amplification

Gene-specific primers used for PCR amplification of *sad1*, *sad2,* *fad2a, fad2b, fad3a* and *fad3b* were designed based on the 5′- and 3′-UTR regions of their Genbank genomic sequences using the Primer3 software (Rozen and Skaletsky 2000) (ESM 2). PCR reactions were carried out in a final volume of 10 μl containing 40 ng of total genomic DNA, 0.4 μM each primer, 1× PCR buffer, 1.5 mM MgCl_2_, 0.8 mM dNTPs, 0.1 μl of 10× BSA (1 mg/ml) and 1 unit Taq DNA polymerase. PCR reactions were performed using the following conditions: an initial denaturation of 4 min at 94 °C followed by 35 cycles at 94 °C for 30 s, 60 °C for 30 s, 72 °C for 1–3 min depending on the target and a final extension of 10 min at 72 °C. A total of 6–12 independent PCR reactions were performed for each target gene of each genotype. The PCR products from each gene/genotype were pooled and aliquots were visualized by agarose gel electrophoresis to verify amplicon specificity.

### DNA sequencing

The pooled PCR amplicons were purified with Multiscreen_384_-PCR filter plates according to the manufacturer’s instructions (Millipore Corp., Billerica, MA, USA). Aliquots of each purified pooled PCR amplicon were resolved on 1 % agarose gels to estimate DNA concentration. Aliquots of the purified PCR amplicons were sequenced with Big-Dye V3.1 Terminator chemistry (Applied BioSystems, Foster City, CA, USA) using amplicon-specific primers designed to span the entire amplicons with overlap in both orientations (ESM 2). Sequencing reactions were performed in a volume of 6 μL containing 40 ng of purified amplicons, 1 μl of 5× sequencing buffer, 8.7 μM primer and 0.4 μl BigDye reaction mix. Reactions were carried out under the following conditions: an initial denaturation of 5 min at 92 °C followed by 60 cycles at 92 °C for 10 s, 55 °C for 5 s, 60 °C for 4 min and a final extension step of 10 min at 60 °C. Unincorporated dideoxynucleotides were removed by ethanol precipitation prior to resolution of the sequences on an ABI 3130xl Genetic Analyzer (Huang and Cloutier 2008).

### Genetic diversity analysis

DNA trace files from the ABI 3130xl Genetic Analyzer were processed and assembled using an internal data pipeline called SOOMOS v0.6 (T. Banks, personal communication) which implements the base calling software PHRED (Ewing et al. 1998) and the assembly software CAP3 (Huang and Madan 1999). Multiple alignment, translation and identification of open reading frames (ORFs) were conducted using clustalW v1.82 (Higgins et al. 1994) and DNAMAN v3.2 (Lynnon Corp., Vaudreuil-Dorion, Quebec, Canada). Assemblies were manually curated to correct sequencing errors.

### Phylogenetic analysis

Phylogenetic analyses were performed using MEGA 4.0 (Tamura et al. 2007). Phylogenetic trees based on the alignment of full-length DNA sequences of each gene were constructed using the neighbor-joining (NJ) algorithm (Saitou and Nei 1987) as implemented in MEGA 4.0. Bootstrap values were estimated using 1,000 replications.

### Field trials and phenotyping of fatty acid compositions

The 120 flax accessions were grown in a type 2 modified augmented design (MAD) (Lin and Poushinsky 1985) at the Kernen farm near Saskatoon (SK, Canada) and at the Morden Research Station (MB, Canada) in 2009, 2010 and 2011. In the MAD, plots were arranged in 10 × 10 grids and each main plot was split into five subplots where the central subplot was occupied by the main plot control cultivar ‘CDC Bethune’. Two additional subplot controls, ‘Macbeth’ and ‘Hanley’, were assigned to two random subplots of five randomly selected whole plots. The 120 flax accessions were randomly allocated to the remaining subplots. The design and assignment of flax accessions were conducted using the Agrobase software (Agronomix Software Inc, Winnipeg, Canada). Oil content (OIL) was determined by nuclear magnetic resonance calibrated against the FOSFA (Federation of Oils, Seeds and Fats Associations Limited) extraction method. Fatty acid profiles were obtained using fatty acids methyl esters (FAMEs) extracted from seeds (AOAC method 996.06) (Daun and Mazur 1983). FA composition of each line was measured on a Varian 3800 gas chromatograph (GC) (Varian Analytical Instruments, Mississauga, ON, Canada). FA compositions were expressed as the relative percentage of PAL, STE, OLE, LIO and LIN. Iodine value (IOD) was calculated from the GC-determined fatty acid composition (AOCS Method Cd 1c-85).

### Statistical analysis

All observed values for FA composition, OIL and IOD obtained from six individual experiments (3 years and two locations; ESM 3) were analyzed individually and adjusted for soil heterogeneity based on the MAD statistical analysis method and pipeline programs described by You et al. (2013). To assess the differences among isoforms, one-way analysis of variance (ANOVA) with unequal sample sizes was used, followed by the Duncan’s multiple range comparison tests at 0.05 probability level. All statistical analyses were carried out using SAS v9.2 (SAS Institute, Cary, USA). The ANOVAs were repeated excluding the seven EMS mutant lines from the data set.

## Results

DNA sequences spanning the entire coding region of *sad1*, *sad2*, *fad2a*, *fad2b*, *fad3a* and *fad3b* from 120 flax genotypes were obtained. BLASTN and BLASTX searches against the NCBI non-redundant (nr/nt) databases were used to identify the coding regions and the open reading frames of each gene. Exon and intron structure were determined and amino acid sequences were deduced. Alleles were numbered and isoforms were identified with letters. The number of alleles ranged from five for *fad2b* to 21 for *fad2a*, and the number of corresponding isoforms ranged from 2 to 7 (Table 1).

**Table 1:** Allelic diversity for six fatty acid desaturases and their deduced isoforms

Gene	Length	Exons	Introns	Exon length	Amino acids	Alleles	Isoforms
*sad1*	2,515	3	2	1,191	396	6	4
*sad2*	2,519	3	2	1,191	396	6	2
*fad2a*	1,137	1	–	1,137	378	21	3
*fad2b*	1,149	1	–	1,149	382	5	4
*fad3a*	3,280	6	5	1,179	392	15	6
*fad3b*	3,002	6	5	1,176	391	18	7

### *Sad1* and *sad2*

**Fig. 1:**
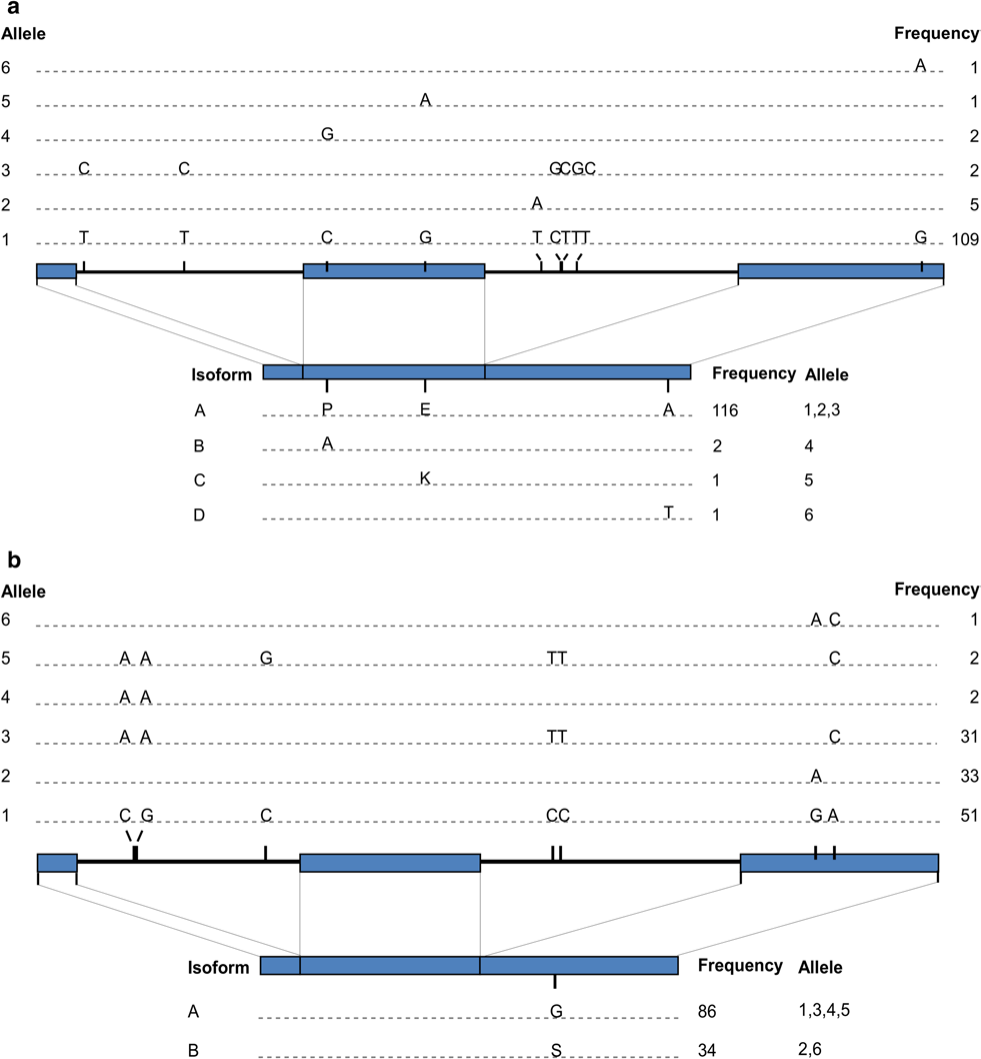

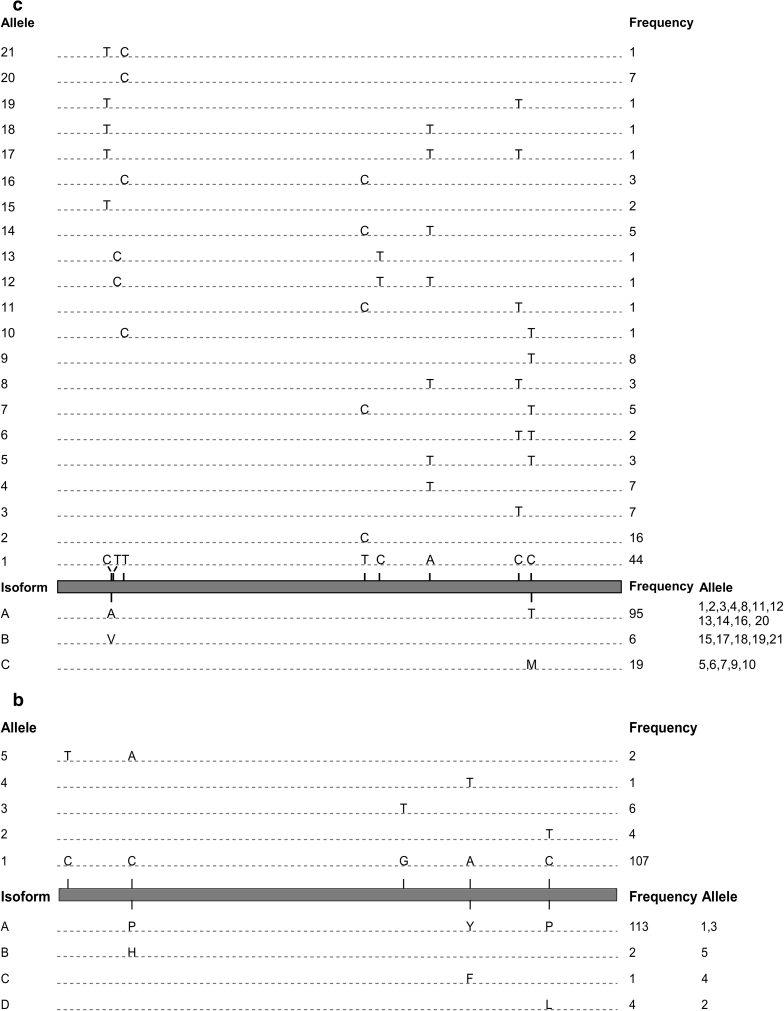

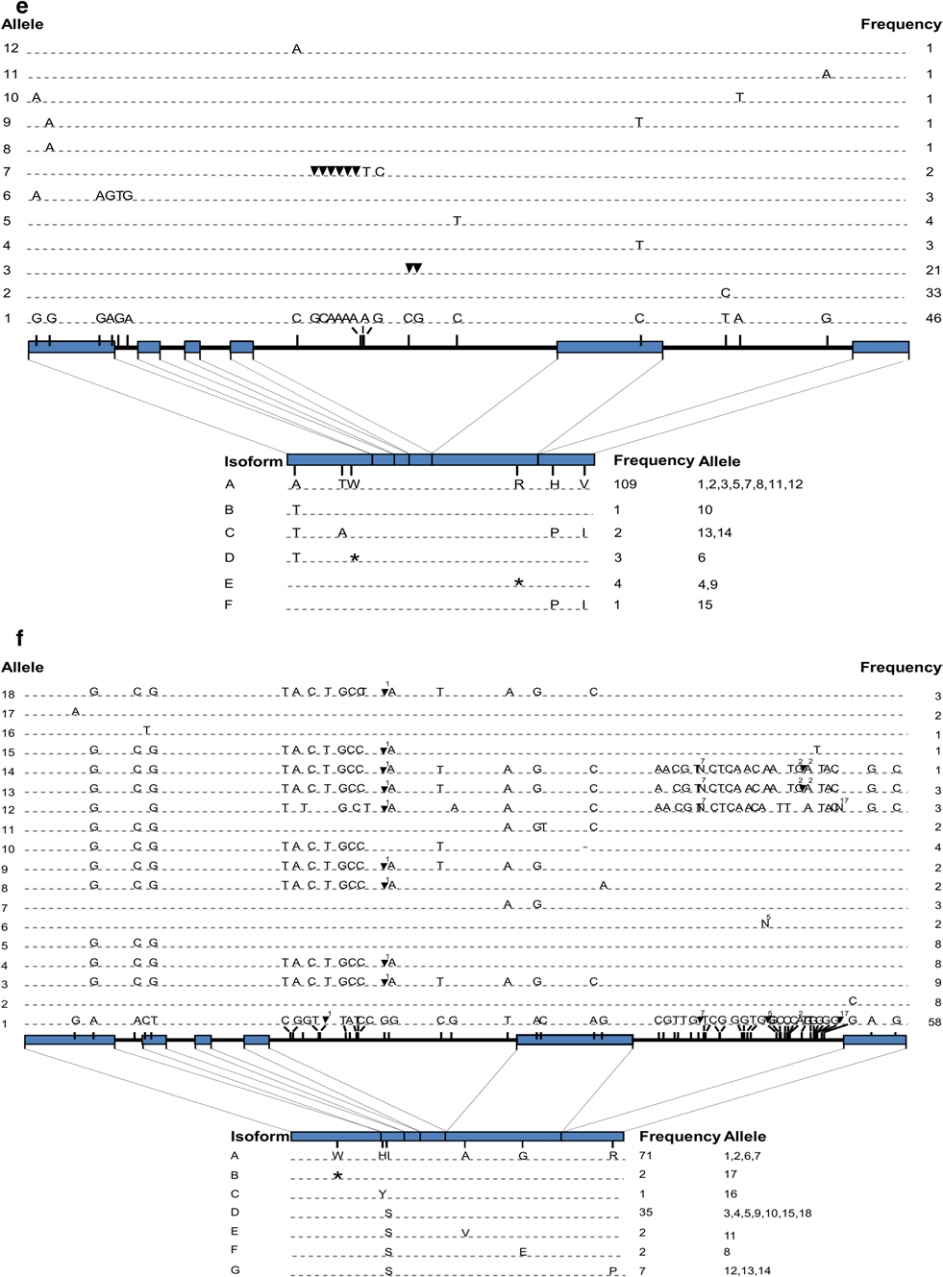
Schematic diagram of alleles and predicted isoforms of six desaturase genes and proteins obtained from sequencing these genes in 120 flax genotypes. SNPs and indels defining the alleles and their frequency in the germplasm are illustrated in the *upper panels*. Amino acid substitutions defining the isoforms, their frequency and corresponding alleles are illustrated in the *lower panels*. Exons are drawn as *boxes* and introns as *lines*. Deletions are represented by *inverted triangles* with the number of bases deleted. **a** *sad1*, **b** *sad2*, **c** *fad2a*, **d** *fad2b,* **e** *fad3a*, **f** *fad3b*. *Fad3a* alleles 13, 14 and 15 are not illustrated because they are hypervariable

The two *sad* genes shared a similar overall structure with three exons and two introns (Fig. 1a, b). The length of the coding region was 2,515 bp for *sad1* and 2,519 bp for *sad2*. Both genes encode proteins of 396 amino acid residues and share 91 % identity at the DNA level and 99 % at the amino acid level (ESM 4). A total of 10 SNPs in the *sad1* coding region defined six alleles (Table 2). Three mutations were missense and seven were silent (Fig. 1a). Allele 1 was found in 109/120 accessions, while the other five alleles were present in only one to five accessions. Alleles 1, 2 and 3 encoded isoform A present in 116 accessions. Alleles 4, 5 and 6 each caused amino acid substitutions resulting in isoform B, C and D, respectively (Fig. 1a). Seven SNPs were identified in *sad2*, thus defining six alleles (Fig. 1b; Table 2). Alleles 1, 2 and 3 were found in 51, 33 and 31 accessions, respectively, while the other three were rare. Of the two SNPs in exon 3, only one caused an amino acid substitution of a glycine to a serine, thus defining isoform A and B, present in 86 and 34 accessions, respectively (Fig. 1b).

**Table 2:** SNPs and indels identified in *sad* and *fad* genes sequenced from 120 flax accessions

Gene	SNP location		SNP frequency (SNP/100 bp)	Indel location		Indel frequency
	Exon	Intron		Exon	Intron	(Indel/100 bp)
*sad1*	3	7	0.40	–	–	–
*sad2*	2	5	0.28	–	–	–
*fad2a*	8	–	0.70	–	–	–
*fad2b*	5	–	0.44	–	–	–
*fad3a*	14	96	3.35	–	19	0.58
*fad3b*	10	40	1.66	–	5	0.17

### *Fad2a* and *fad2b*

The *fad2a* and *fad2b* intron-less genes spanned 1,137 and 1,149 bp encoding proteins of 378 and 382 amino acid residues, respectively (Fig. 1c, d). The two sequences shared 82 % identity at the DNA level and 87 % at the amino acid level (ESM 5). Of the six desaturase genes sequenced herein, the *fad2a* gene had the most alleles with 21 as defined by eight SNPs (Table 2). The most frequent allele was present in 44 accessions, while the remaining 20 were found in 1–16 accessions (Fig. 1c). Despite the high allelic variation, only three isoforms of FAD2A were deduced because only two of the eight SNPs were non-synonymous. A total of five SNPs formed the five *fad2b* alleles, of which three were non-synonymous, hence the four FAD2B isoforms (Fig. 1d; Table 2).

### *Fad3a* and *fad3b*

The coding regions of *fad3a* and *fad3b* were 3,280 and 3,002 bp, respectively, both encompassing six exons and five introns (Fig. 1e, f). The deduced protein sequences had 392 and 391 amino acid residues. The two sequences displayed only 85 % identity at the DNA level, but 94 % at the amino acid level (ESM 6). Indels located in introns and ranging from 1 to 29 bp were responsible for the 278 bp length difference and divergence between the two *fad3* paralogs (Table 2). Because three of the *fad3a* alleles were hypervariable, the assembly of their coding sequences yielded two distinct contigs. The first contig included the *fad3a* sequences from 117 accessions (Fig. 1e), while the remaining three formed the second contig (ESM 7). Taken together, 110 SNPs and 19 indels were identified in this gene. However, the majority were the results of the hypervariable alleles 13, 14 and 15 as only 14 SNPs and two indels were detected in the remaining 12 alleles. Considering all 120 *fad3a* sequences, only 14 SNPs and no indels were detected in exons, thereby encoding six isoforms, of which, isoform A was present in 109 accessions. The hypervariable alleles 13, 14 and 15 were predicted to encode two different isoforms (C and F). Isoform D and isoform E had premature stop codons, a feature not observed in *sad* and *fad2* genes.

Fifty SNPs and five indels defined 18 different alleles of *fad3b* (Table 2). The most common allele was present in nearly half of the accessions. Among the 50 SNPs, only six were non-synonymous (Fig. 1f). A transition from G to A in the first exon of allele 17 resulted in a nonsense mutation leading to a stop codon near the N-terminus of the FAD3B protein, which is predicted to encode a truncated desaturase of 53 amino acids. Another single-point mutation, identified in the second exon, was responsible for a histidine to tyrosine substitution in the first HIS-box (isoform C). Isoforms D, E, F and G all shared an isoleucine to serine substitution, but isoform E, F and G each had one additional but different amino acid substitution (Fig. 1f).

### Phylogeny of desaturases genes

The neighbor-joining trees of *sad1* and *sad2* had very similar topology with the clustering of each of the six alleles into two major clades (ESM 8). NJ trees derived from *fad2a* and *fad2b* sequences differed significantly in their topology (ESM 9). The *fad2a* sequences formed four distinct clades including a number of sub-clades showing a higher sequence divergence among *fad2a* alleles. In contrast, the *fad2b* NJ tree showed a substantially reduced nucleotide diversity among alleles, in which four of the five alleles grouped together to form clade II (ESM 9). NJ trees of *fad3a* and *fad3b* had two distinct clades with higher bootstrap support. However, the *fad3a* NJ tree showed less divergence between alleles with the exception of the hypervariable alleles which grouped together to form clade I. In contrast, the *fad3b* NJ tree showed significant sequence divergence between alleles implying higher accumulation of mutations through evolution (ESM 10).

### Association between fatty acid composition and desaturase isoforms

**Fig. 2:**
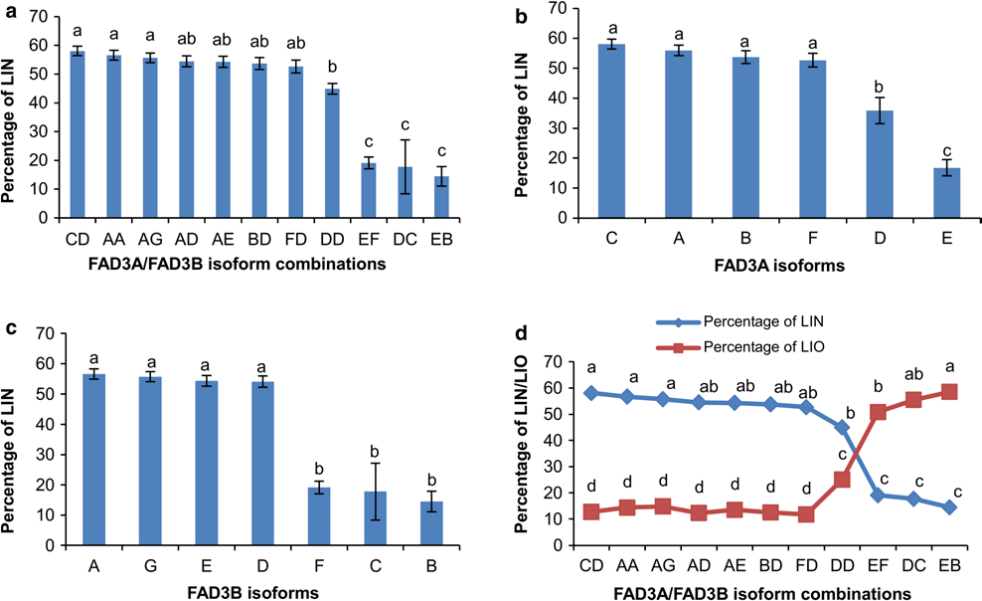
Association between the predicted isoforms of **a** FAD3A/B and LIN content, **b** FAD3A and LIN content, **c** FAD3B and LIN content and **d** FAD3A/B and LIN and LIO contents. *Vertical bars* represent standard error of the mean. *Letters on top of the bar* indicate statistical significance of Duncan’s multiple range tests

A one-way ANOVA was conducted to compare the effect of the predicted isoforms of SAD, FAD2 and FAD3, individually and in combinations on the fatty acid composition, OIL and IOD. Although lines carrying SAD2 isoform B accumulated significantly more OLE, all SAD combinations and the two SAD1 isoforms individually had no significant effect on OLE (ESM 11). However, the effect of SAD1/2 combinations was significant on STE (Table 3). Significant differences were observed between FAD2A/B combinations for OLE and OIL content and FAD2A isoforms for LIO and OIL content. Among the three different FAD2A isoforms, lines carrying isoform C accumulated significantly more LIO (ESM 12). FAD3A/B isoform combinations were significant for LIN, LIO, OLE, PAL, OIL and IOD traits. FAD3A and FAD3B isoforms were also individually significant for LIN, LIO, PAL, OIL and IOD with FAD3A not being significant for OLE acid (Table 3). Lines carrying FAD3A isoforms D and E and FAD3B isoforms B, C and F individually as well as in combinations (EF, DC and EB) accumulated significantly less LIN (Fig. 2a–c). LIO content was significantly elevated in lines having the EF, DC and EB combinations revealing a strong inverse association between LIN and LIO (Fig. 2d).

**Table 3:** Effect of SAD and FAD predicted isoforms on palmitic, stearic, oleic, linoleic and linolenic acid composition, oil content and iodine value

Trait	Gene	*p* value	Predicted isoforms or combinations^†^
Palmitic acid (PAL)	*sad1* *sad2* *fad2a* *fad2b*	0.8225 0.6520 0.1886 0.8978	
	*fad3a*	<.0001*	[E(7.84)]^a^, [A,B,C,D,F(5.96–5.59)]^b^
	*fad3b*	<.0001*	[F(9.42)]^a^, [A,B,C,D,E,G(6.38–5.48)]^b^
	*sad1/sad2* *fad2a/fad2b*	0.8547 0.6547	
	*fad3a/fad3b*	<.0001*	[EF(9.42)]^a^, [AA,AD,AE,AG,BD,CD,DC,DD,EB,FD(6.37–5.48)]^b^
Stearic acid (STE)	*sad1*	0.1731	
	*sad2*	0.0832	
	*fad2a*	0.2950	
	*fad2b*	0.1558	
	*fad3a*	0.8507	
	*fad3b*	0.2350	
	*sad1/sad2*	0.0425*	[CB(6.62)]^a^, [DB(4.60)]^ab^, [AA, AB,BB,(4.29–3.78)]^b^
	*fad2a/fad2b*	0.3162	
	*fad3a/fad3b*	0.4552	
Oleic acid (OLE)	*sad1*	0.9703	
	*sad2*	0.0083*	[B(21.64)]^a^, [A(19.71)]^b^
	*fad2a*	0.0067*	[A(20.79)]^a^, [C(18.35)]^ab^, [B(17.90)]^b^
	*fad2b*	0.3225	
	*fad3a*	0.2275	
	*fad3b*	0.0009*	(16.48–22.46)^a^
	*sad1/sad2*	0.0944	
	*fad2a/fad2b*	0.0054*	(15.59–20.99)^a^
	*fad3a/fad3b*	0.0031*	[FD(24.18)]^a^, [BD(23.77)]^ab^, [AA,AD,AE,AG,CD,DD(22.79–19.23)]^abc^, [DC(17.08)]^bc^, [EB,EF(16.78–16.46)]^c^
Linoleic acid (LIO)	*sad1*	0.9220	
	*sad2*	0.7126	
	*fad2a*	0.0076*	[C(21.45)]^a^, [A,B(14.76–12.91)]^b^
	*fad2b*	0.9755	
	*fad3a*	<.0001*	[E(54.67)]^a^, [D(35.23)]^b^, [A,B,C,F(13.88–11.76)]^c^
	*fad3b*	<.0001*	[B(58.52)]^a^, [C(55.49)]^ab^, [F(50.81)]^b^,[A,D,E,G(14.87–13.06)]^c^
	*sad1/sad2*	0.9722	
	*fad2a/fad2b*	0.1268	
	*fad3a/fad3b*	<.0001*	[EB(58.52)]^a^, [DC(55.49)]^ab^, [EF(50.81)]^b^, [DD(25.11)]^c^, [AA,AD,AE,AG,BD,CD,FD(14.87–11.76)]^d^
Linolenic acid (LIN)	*sad1*	0.9643	
	*sad2*	0.6721	
	*fad2a*	0.0707	
	*fad2b*	0.7270	
	*fad3a*	<.0001*	[A,B,C,F(58.08–52.70)]^a^, [D(35.86)]^b^, [E(16.80)]^c^
	*fad3b*	<.0001*	[A,D,E,G(56.64–54.07)]^a^, [B,C,F(19.12–14.48)]^b^
	*sad1/sad2*	0.9596	
	*fad2a/fad2b*	0.3172	
	*fad3a/fad3b*	<.0001*	[AA,CD,AG(58.08–55.70)]^a^, [AD, AE,BD,FD(54.484–52.70)]^ab^, [DD(44.91)]^b^, [DC,EB,EF(19.12–14.48)]^c^
Oil content (OIL)	*sad1*	0.9779	
	*sad2*	0.9024	
	*fad2a*	0.0004*	[A,C(44.14–42.80)]^a^, [B(39.90)]^b^
	*fad2b*	0.2108	
	*fad3a*	0.0044*	[C,D(47.49–45.48)]^a^, [A,B,E(43.18–42.71)]^ab^, [F(39.68)]^b^
	*fad3b*	0.0136*	[C(48.46)]^a^,[A,B,D,E,F,G(43.81–42.31)]^b^
	*sad1/sad2*	0.9955	
	*fad2a/fad2b*	0.0068*	[CA(44.20)]^a^, [AA,AB,AD,BA,CD(43.10–40.17)]^ab^, [BC(38.52)]^b^
	*fad3a/fad3b*	0.0056*	[DC(48.46)]^a^, [CD,DD(47.00–45.48)]^ab^, [AD,AE,EF(43.81–43.54)]^abc^, [AA,AG,BD,EB(42.85–42.31)]^bc^, [FD(39.68)]^c^
Iodine value (IOD)	*sad1* *sad2* *fad2a* *fad2b*	0.9949 0.4642 0.3869 0.3647	
	*fad3a*	<.0001*	[C(190.68)]^a^, [A,B,F(187.97–179.11)]^ab^, [D(170.86)]^b^, [E(152.96)]^c^
	*fad3b*	<.0001*	[A,E,D,G(189.88–183.40)]^a^, [C,B,F(157.33–152.15)]^b^
	*sad1/sad2* *fad2a/fad2b*	0.9516 0.3689	
	*fad3a/fad3b*	<.0001*	[AA,AD,AE,AG,BD,CD,DD,FD(190.68–177.63)]^a^, [DC,EB,EF(157.33–152.15)]^b^

* Statistical significance (*p* < 0.05)

^†^Means for the isoform(s) or isoform combinations are in bracket. They represent data collected from two locations during three years

^a, b, c^ Statistical significance of Duncan’s multiple range tests

We suspected that some of the significance was attributed to non-functional isoforms exclusively found in EMS mutant lines. To test this hypothesis, we repeated the one-way ANOVA using only the 113 non-mutant lines. This had the effect of eliminating isoforms D and E for FAD3A, and B, C and F for FAD3B. Overall, fewer desaturases were significant for most of the traits, but several significant associations found with the whole dataset remained significant with the reduced dataset (ESM 13). Of particular interest are the significant associations between FAD3B isoforms and PAL, SAD2 and FAD2A isoforms and OLE, and FAD3B isoforms, and LIO. Also, OIL was significantly affected by FAD2A isoforms and FAD2A/B isoform combinations.

## Discussion

FA desaturases introduce double bonds at specific locations of fatty acid acyl chains and, hence, are considered targets for manipulation of fatty acid composition of oilseed crops. FA desaturases exhibit significant diversity in their sequences and expression (Los and Murata 1998; Warude et al. 2006). Minor changes in the primary structure of proteins may result in modification of the enzyme function including altered substrate specificity, region selectivity or loss of function (Avelange-Macherel et al. 1995; Broadwater et al. 2002; Khadake et al. 2011). In the present study, the extent of the genetic variability for six desaturase genes (*sad1*, *sad2*, *fad2a*, *fad2b*, *fad3a* and *fad3b*) was determined by sequencing them from 120 genotypes of flax. DNA sequences were obtained to quantify the scope of the genetic variations and to predict structural changes of the encoded desaturases. These genotypic data were correlated to the fatty acid composition obtained from multi-year, multi-location field-grown material to determine the functional role of these alleles and isoforms on fatty acid composition and oil content.

Sequence analysis of the six genes revealed a significant level of variation at the nucleotide level with SNPs being the most frequently observed mutation type. Most of these point mutations were synonymous substitutions that did not alter the underlying amino acid sequences. SNPs are the most abundant type of DNA variation of plant genomes (Brookes 1999; Wei et al. 2011). However, their frequency varies among the different plant species (Wei et al. 2011). Inbred rice and *Arabidopsis* display one SNP in every 300 bp (Schmid et al. 2003), while outbreeding maize has one SNP/60 bp (Ching et al. 2002). This higher SNP rate was observed in *fad3b*, while the rate of *fad3a* was twice as high with one SNP every 30 bp. Nucleotide variations in exons were lower than in introns and most were missense or silent mutations. Both SNPs and indels were present in introns, whereas exons contained only SNPs (Table 2). Exons are under stronger selection pressure resulting in a slower mutation rate caused by the elimination of deleterious mutations from the gene pool (Wei et al. 2011; Gaut 1998).

Plant membrane-bound desaturases are characterized by the presence of three highly conserved HIS-box motifs essential for enzyme activity (Shanklin et al. 1994; Los and Murata 1998). These motifs are involved in the formation of di-ion active sites (Fox et al. 1993 and Shanklin et al. 1994). Consistent with other plant desaturases, the four membrane-bound *fad* genes sequenced herein are predicted to encode FADs with the highly conserved histidine-rich motifs. Only accession SP2047 had a point mutation in one of the HIS-box of FAD3B which was previously shown to be non-functional (Banik et al. 2011). The level of sequence conservation observed between the paralogous desaturases was also reported for other plant desaturases (Scheffler et al. 1997). *Sad1* and *sad2* have the most highly conserved exon structure. Several SADs have been cloned and characterized from various crops such as castor bean, soybean, safflower, *Arabidopsis* and flax (Jain et al. 1999; Singh et al. 1994; Knutzon et al. 1991; Shanklin and Somerville 1991). The high sequence identity at both DNA and amino acid level between *sad* sequences was reported in other plants (Browse and Somerville 1991; Luo et al. 2009; Shanklin and Somerville 1991; Singh et al. 1994) and can be interpreted as an indication of the essential role of ∆9-desaturase in lipid biosynthetic pathway in plants.

C18 unsaturated fatty acids of the plastid and the microsomal membranes originate from the desaturation of stearoyl-ACP in the plastid by SAD, thus serving as an attractive target for altering the unsaturated fatty acid content in oil crops (Ohlrogge and Jaworski 1997). Modification in the synthesis of C18 unsaturated fatty acids may impair membrane fluidity, because they are part of structural membranes as well as major components of seed storage oil (Lightner et al. 1994). The complexity of the multi-gene *sad* family is another indication of its essential role in plants (Ohlrogge and Jaworski 1997; Jain et al. 1999; Fofana et al. 2006). The more conserved nature of *sad2* with a few nucleotide changes is supported by previous studies showing stronger expression of *sad2* in flax (Allaby et al. 2005). The *sad2* locus appeared to be the more physiologically important of the two. The significant effect of SAD2 isoforms on the OLE content corroborates this assumption.

Unsaturated fatty acids in plants play essential roles in membrane integrity and function, cellular signaling, thermal adaptation and energy storage (Browse and Somerville 1991; Mikami and Murata 2003; Zhang et al. 2012). Desaturation of OLE into LIO is considered an important step affecting the quality of seed storage oils, as it initiates the synthesis of PUFAs from monounsaturated OLE. Although a single *fad2* gene was identified in *Arabidopsis*, this gene appears to exist as a gene family in most other plants including flax, soybean, cotton and safflower (Fofana et al. 2004; Krasowska et al. 2007; Khadake et al. 2009; Heppard et al. 1996; Li et al. 2007; Zhang et al. 2009; Cao et al. 2013). Recent studies have demonstrated that genetic variation in *fad2* was associated with consequent changes in fatty acid profiles (Pham et al. 2011; Wang et al. 2011). *Fad2* genes are thought to be rate limiting in fatty acid biosynthesis pathway in flax and are highly influenced by the environment (Fofana et al. 2006). *Fad2a*’s highest allelic diversity of 21 alleles contrasted with the conservation of *fad2b* with only 5. The *fad2b* gene plays a major role in producing LIO and remains a housekeeping microsomal Δ12 oleate desaturase with constitutive expression throughout the plant (Cao et al. 2013; Schlueter et al. 2007). In soybean, *fad2*-*2*, the ortholog of *fad2b*, was shown to be the most important gene for increasing LIO content (Schlueter et al. 2007). Site-directed mutagenesis of *fad2*, altering a few amino acid residues, modified the enzymatic activity of the encoded FAD2 in *Lesquerella fendleri* and *Synechocystis* sp. (Avelange-Macherel et al. 1995; Broun et al. 1998). Similarly, through our characterization of the *fad2* genetic variability and its correlation with fatty acid composition, we identified the FAD2A-C isoform to be correlated with a significantly higher level of LIO, suggesting a positive effect of the threonine to methionine substitution located in the vicinity of the third HIS-box.

Two distinct pathways operating in the plastids and the endoplasmic reticulum are responsible for the synthesis of C18:3 fatty acids in plants. Several endoplasmic *fad3* genes have been cloned and characterized from various plants such as *Brassica*, safflower, flax and *Arabidopsis* (Arondel et al. 1992; Yadav et al. 1993; Vrinten et al. 2005). Flax displays wide genetic variability for LIN content with traditional linseed varieties having 50–59 % linolenic acid, high-LIN varieties with 60–70 % (Friedt et al. 1995; Kenaschuk 2005) and Solin varieties with 2–4 %. The first Solin lines were developed using mutation breeding of flax varieties Glenelg and McGregor (Green 1986a; Rowland 1991). As revealed by sequence analysis, the *fad3* genes were hypervariable with numerous SNPs and indels. *Fad3* genes also carried nonsense mutation resulting in premature stop codons, a feature only observed in induced mutants or lines derived from them. Naturally occurring allelic diversity in plants has been considered an important genetic factor for phenotypic variation (Buckler and Thornsberry 2002). Induced mutations eliminate or cause a large reduction of a functional gene product, whereas naturally occurring allelic variation alters the gene products and may be the fundamental mechanism for quantitative trait variation (Yano and Sasaki 1997; Mackay 2001). Similarly, the portion of the allelic diversity and novel isoforms discovered in induced mutant lines were not present in the natural germplasm. EMS mutant lines (Double Low, UGG146-1, SP2047, E1747, YSED18, M96006 and S95407) carrying stop codons in the FAD3A-D, FAD3A-E and FAD3B-B isoforms and HIS-box mutation in the FAD3B-C isoform accumulated significantly reduced levels of LIN. The inability of the mutated FAD3 to perform the desaturation of LIO to LIN in induced mutant lines is supported by previous studies showing additive gene effects across the two loci on desaturation of LIO into LIN (Green 1986b) and the impaired biochemical activity of the mutant alleles (Stymne et al. 1992). These studies validate the conclusion that the mutant *fad3* alleles either producing truncated proteins or carrying HIS-box mutations are inactive.

The amino acid substitutions in FAD3B-D and -F isoforms may also have a negative influence on FAD3B activity. FAD3A and FAD3B have been shown to be the major enzymes controlling the LIN content in flaxseeds (Vrinten et al. 2005). Vrinten et al. (2005) showed that both *fad3a* and *fad3b* carried point mutations leading to premature stop codons in line 593–708, resulting in 2–3 % LIN content. Similarly, HIS-box mutation in *fad3b* gene in Solin line SP2047 caused enzyme inactivity (Banik et al. 2011).

The significant inverse relationship of LIN content with LIO found in this study was also reported in a number of crops including flax, soybean and almonds (Wakjira et al. 2004; Thomas et al. 2003; Abdallah et al. 1998). This inverse association is in agreement with the fact that the biosynthesis of LIN occurs through the stepwise desaturation of OLE via LIO (Ayerza 2009). Thus, LIO accumulates in FAD3 mutant lines (Bocianowski et al. 2012). Since IOD measures the degree of unsaturation, lines with elevated LIN content also show higher IOD (Cloutier et al. 2010). Several studies have demonstrated the correlation between oil content and levels of saturated fatty acids (Velasco et al. 2007). An increase in PAL by 1 % led to a decrease in oil content of 1.4 % in rapeseed (Mollers and Schierholt 2002). In soybean, both mutants with reduced and elevated PAL led to a decrease in seed oil content in comparison with lines with standard fatty acid composition (Ndzana et al. 1994; Hartmann et al. 1996; Stoltzfus et al. 2000). However, the correlation between oil content and the levels of unsaturated fatty acids has not been fully elucidated. While a few studies found no adverse effect of high oleic acid soybeans with 80 % OLE content on yield and oil content (Kinney 1996; Graef et al. 2009), Brace et al. (2011) showed a statistically significant reduction in both oil content and yield in high oleic acid soybeans. The significant differences observed in OIL content with respect to FAD2A/B and FAD3A/B isoform combinations are not consistent with elevated or reduced LIO or LIN content, suggesting further studies to elucidate correlations. However, seed oil content is a complex quantitative trait governed by a number of genes and also influenced by the environment (Burton 1987; Cloutier et al. 2010; Lee et al. 2007; Eskandari et al. 2013). Therefore, it is likely that these correlations may not only be determined by genetic factors, but also influenced by the environment.

Genetic redundancy drives evolution by allowing functional diversification while simultaneously retaining the original function(s) of essential genes (Cao et al. 2013). In flax, the SAD, FAD2 and FAD3 enzymes are encoded by duplicated genes (Fofana et al. 2010). Following duplication, paralogs can retain their original gene function, gain new function(s) or be silenced (Force et al. 1999). In flax, functional redundancy of six paralog desaturases provides additional buffering capacity for mutation tolerance even in exons as exemplified by the predicted non-functional FAD3A-D, FAD3A-E and FAD3B-B. A duplicated pair of genes can have an altered selective pressure, leading to the loss of one copy or to an increased rate of divergence in sequence when both copies are preserved (Fischer et al. 2001). Here, *fad3a* and *fad3b* seem to be functionally preserved and the redundancy may have allowed for the higher divergence between sequences, consistent with previous studies showing the additive role of *fad3a* and *fad3b* and their equal contribution to LIN content in flax (Vrinten et al. 2005; Banik et al. 2011).

Selection pressure plays a prominent role in decreasing nucleotide diversity in domesticated crops (Wei et al. 2011). In flax, the overall reduction of nucleotide diversity during domestication is at a moderate level (27 % with respect to pale flax) when compared with other inbred species such as wheat and barley (Fu 2011). Selection pressure over the process of domestication might have a significant impact on the observed variation in *fad2,* *sad1* and *sad2* as illustrated by the NJ trees (EMS8, 9, 10). The impact of selection on *fad2* and *fad3* diversity during domestication was also reported in cultivated sunflower (Chapman and Burke 2012).

FA composition can be altered by manipulating one or more steps of their biosynthesis pathway (Ohlrogge and Jaworski 1997; Thelen and Ohlrogge 2002; Cahoon et al. 2010). Most domesticated oilseed crops have been modified to obtain optimized FA profiles providing specific end uses through approaches such as classical breeding or genetic engineering (Drexler et al. 2003). Suppression of the ∆12-desaturase gene in soybean, sunflower, cotton and canola has successfully increased OLE content in their seed oils (Metzger and Bornscheuer 2006). Novel allelic variants and isoforms identified for the six desaturases provide useful genetic and molecular resources and information for the development of oilseed flax with unique and useful oil profiles that would not require a transgenic or mutagenesis approach.

## Electronic supplementary material

Below is the link to the electronic supplementary material.

Supplementary material 1 (PDF 285 kb)

Supplementary material 2 (PDF 113 kb)

Supplementary material 3 (PDF 315 kb)

Supplementary material 4 (PDF 104 kb)

Supplementary material 5 (PDF 146 kb)

Supplementary material 6 (PDF 156 kb)

Supplementary material 7 (PDF 91 kb)

Supplementary material 8 (PDF 21 kb)

Supplementary material 9 (PDF 175 kb)

Supplementary material 10 (PDF 26 kb)

Supplementary material 11 (PDF 10 kb)

Supplementary material 12 (PDF 11 kb)

Supplementary material 13 (PDF 96 kb)
