# Supplementary material for: Genetic variation of six desaturase genes in flax and their impact on fatty acid composition
Source: Theor Appl Genet. 2013 Aug 9;126(10):2627–41. doi: 10.1007/s00122-013-2161-2 (PMC3782649; doi:10.1007/s00122-013-2161-2)
Supplement: Supplementary file 1 — Supplementary material 1 (PDF 285 kb) [file 122_2013_2161_MOESM1_ESM.pdf]

Table S1. List of accessions from which the six desaturase genes were sequenced

Accession/Description	Accession number	Species/type[2]	Origin[3]	*sad* allele and isoform				*fad2* allele and isoform				*fad3* allele and isoform			
				sad1		***sad2***		***fad2a***		***fad2b***		***fad3a***		***fad3b***	
AC Watson	CN18973	*L. usitatissimum*/O	CAN	1	A	1	A	11	A	1	A	1	A	2	A
Flanders	CN18979	*L. usitatissimum*/O	CAN	1	A	3	A	3	A	1	A	3	A	1	A
Somme	CN18980	*L. usitatissimum*/O	CAN	1	A	1	A	1	A	1	A	3	A	2	A
CDC Valour	CN18981	*L. usitatissimum*/O	CAN	1	A	2	B	2	A	1	A	1	A	2	A
Evelin	CN18982	*L. usitatissimum*/F	FRA	1	A	1	A	2	A	1	A	1	A	1	A
Laura	CN18983	*L. elongatum*/F	NLD	1	A	1	A	1	A	1	A	1	A	1	A
Hermes	CN18986	*L. usitatissimum*/F	FRA	1	A	2	B	1	A	1	A	2	A	1	A
Viking	CN18987	*L. elongatum*/F	NLD	1	A	2	B	1	A	1	A	2	A	1	A
Ariane	CN18988	*L. elongatum*/F	FRA	1	A	2	B	2	A	1	A	1	A	1	A
Atalante	CN18989	*L. usitatissimum*/O	FRA	1	A	1	A	7	C	1	A	3	A	1	A
Nike	CN18991	*L. elongatum*/F	POL	1	A	1	A	1	A	1	A	1	A	1	A
Linda	CN18993	*L. usitatissimum*/O	NLD	1	A	1	A	1	A	1	A	2	A	4	D
Verne	CN18994	*L. usitatissimum*/O	USA	1	A	2	B	1	A	1	A	3	A	1	A
Raisa	CN18997	*L. elongatum*/F	NLD	1	A	1	A	2	A	1	A	1	A	4	D
Escalina	CN18998	*L. elongatum*/F	NLD	1	A	1	A	1	A	1	A	2	A	1	A
Marina	CN19001	*L. elongatum*/F	NLD	1	A	1	A	1	A	1	A	2	A	1	A
AC McDuff	CN19003	*L. usitatissimum*/O	CAN	1	A	2	B	5	C	1	A	3	A	1	A
AC Emerson	CN19004	*L. usitatissimum*/O	CAN	1	A	2	B	1	A	1	A	2	A	1	A
AC Linora	CN19005	*L. usitatissimum*/O	CAN	1	A	1	A	1	A	1	A	1	A	1	A
no name	CN19007	*L. usitatissimum*/O	ETH	1	A	1	A	12	A	1	A	1	A	15	D
CDC Normandy	CN19017	*L. usitatissimum*/O	CAN	1	A	1	A	1	A	1	A	3	A	2	A
Ottawa 829-C	CN19157	*L. elongatum*/O	CAN	1	A	1	A	8	A	2	D	1	A	1	A
Ottawa 770B	CN19158	*L. usitatissimum*/O	CAN	1	A	1	A	1	A	2	D	1	A	13	G
Diadem	CN19159	*L. usitatissimum*/O	CAN	1	A	2	B	1	A	1	A	12	A	1	A
Bolley Golden	CN19160	*L. elongatum*/O	USA	1	A	1	A	1	A	1	A	2	A	1	A
Kirovogradskij 71	CN30860	*L. usitatissimum*/O	UKR	1	A	4	A	14	A	1	A	1	A	5	D
Kubanskij	CN30861	*L. usitatissimum*/O	UNK	1	A	3	A	13	A	1	A	1	A	1	A
Vniil-17	CN32542	*L. elongatum*/F	RUS	1	A	1	A	1	A	1	A	3	A	2	A
Korostenskij 3	CN32546	*L. elongatum*/F	UKR	1	A	1	A	3	A	1	A	1	A	1	A
Linott	CN33385	*L. usitatissimum*/O	CAN	1	A	2	B	4	A	1	A	1	A	2	A
Noralta	CN33386	*L. usitatissimum*/O	CAN	1	A	1	A	5	C	1	A	1	A	1	A
Redwood 65	CN33388	*L. usitatissimum*/O	CAN	1	A	1	A	5	C	1	A	3	A	1	A
Rocket	CN33389	*L. usitatissimum*/O	CAN	1	A	1	A	6	C	1	A	3	A	1	A
Natasja	CN33390	*L. elongatum*/F	NLD	1	A	1	A	3	A	1	A	2	A	1	A
Domtar Selection	CN33393	*L. elongatum*/F	UNK	1	A	1	A	4	A	1	A	1	A	1	A
Dufferin	CN33397	*L. usitatissimum*/O	CAN	1	A	1	A	1	A	1	A	3	A	1	A
Bison	CN33399	*L. usitatissimum*/O	USA	1	A	2	B	1	A	1	A	3	A	1	A

Accession/Description	Accession number	Species/type[2]	Origin[3]	***sad* allele and isoform**				***fad2* allele and isoform**				***fad3* allele and isoform**			
				sad1		***sad2***		***fad2a***		***fad2b***		***fad3a***		***fad3b***	
Norstar	CN33400	*L. elongatum*/O	USA	1	A	2	B	9	C	2	D	3	A	13	G
Culbert	CN33992	*L. usitatissimum*/O	USA	1	A	3	A	9	C	1	A	3	A	1	A
Tverca	CN35791	*L. elongatum*/F	RUS	1	A	1	A	1	A	1	A	1	A	1	A
McGregor	CN37286	*L. elongatum*/O	CAN	1	A	3	A	9	C	1	A	1	A	1	A
Natasja	CN40081	*L. usitatissimum*/F	NLD	1	A	1	A	1	A	1	A	2	A	1	A
Norlin	CN52732	*L. usitatissimum*/O	CAN	1	A	1	A	2	A	1	A	3	A	2	A
Clli-642	CN96845	*L. usitatissimum*/O	RUS	1	A	3	A	21	B	1	A	2	A	1	A
Clli-643	CN96846	*L. usitatissimum*/O	RUS	1	A	2	B	20	A	1	A	13	C	10	D
Clli-1407	CN96911	*L. usitatissimum*/O	TUR	1	A	1	A	4	A	1	A	1	A	11	E
Clli-1455	CN96958	/O	TUR	1	A	3	A	2	A	1	A	2	A	18	D
Clli-1458	CN96962	*L. usitatissimum*/O	TUR	1	A	2	B	16	A	1	A	2	A	10	D
Clli-1470	CN96974	/O	IND	1	A	2	B	2	A	1	A	5	A	3	D
Clli-1499	CN96988	*L. usitatissimum*/O	ETH	1	A	3	A	19	B	1	A	2	A	1	A
Clli-1502	CN96991	*L. usitatissimum*/O	ETH	1	A	3	A	18	B	4	C	2	A	6	A
Clli-1503	CN96992	*L. usitatissimum*/O	ETH	1	A	3	A	17	B	1	A	2	A	6	A
Clli-1519	CN97004	*L. usitatissimum*/O	ETH	1	A	3	A	15	B	1	A	2	A	1	A
Clli-1924	CN97050	*L. usitatissimum*/O	IRN	1	A	2	B	1	A	3	A	1	A	3	D
Clli-1930	CN97056	*L. usitatissimum*/O	PAK	1	A	2	B	2	A	3	A	2	A	3	D
Clli-1938	CN97064	*L. usitatissimum*/O	PAK	1	A	2	B	2	A	3	A	1	A	3	D
Clli-1946	CN97072	/O	PAK	1	A	3	A	1	A	1	A	2	A	3	D
Clli-1957	CN97083	/O	PAK	1	A	2	B	2	A	3	A	1	A	3	D
Clli-1991	CN97092	*L. usitatissimum*/O	PAK	1	A	5	A	1	A	3	A	1	A	3	D
Clli-1995	CN97096	*L. usitatissimum*/O	PAK	1	A	2	B	8	A	3	A	1	A	9	D
Clli-2002	CN97103	*L. usitatissimum*/O	PAK	1	A	2	B	3	A	1	A	1	A	9	D
Clli-2028	CN97129	*L. usitatissimum*/O	IRN	4	B	2	B	3	A	1	A	2	A	10	D
Clli-2028B	CN97129B	/O	IRN	4	B	2	B	4	A	1	A	2	A	10	D
Clli-2038	CN97139	*L. usitatissimum*/O	IRN	1	A	3	A	14	A	1	A	5	A	3	D
Clli-2046	CN97147	*L. usitatissimum*/O	TUR	1	A	1	A	1	A	1	A	11	A	14	G
Clli-2052	CN97153	*L. usitatissimum*/O	TUR	6	D	6	B	1	A	1	A	2	A	11	E
Horal	CN97176	*L. usitatissimum*/O	CZE	1	A	3	A	2	A	1	A	5	A	18	D
Sorth Behbehan	CN97180	*L. elongatum*/F	IRN	1	A	1	A	2	A	1	A	1	A	4	D
noname	CN97214	*L. usitatissimum*/O	ARG	1	A	1	A	1	A	1	A	1	A	1	A
No. 1048	CN97238	*L. usitatissimum*/O	HUN	1	A	1	A	20	A	1	A	1	A	12	G
Lina Deta	CN97287	*L. usitatissimum*/O	HUN	1	A	1	A	16	A	1	A	10	B	4	D
Raja	CN97300	*L. elongatum*/O	HUN	1	A	2	B	2	A	5	B	3	A	12	G
N.P. (R.R.) 9	CN97306	*L. mediterraneum*/O	IND	1	A	3	A	1	A	1	A	2	A	1	A
N.P. (R.R.) 37	CN97307	*L. mediterraneum*/O	IND	1	A	3	A	1	A	1	A	2	A	5	D
N.P. (R.R.) 38	CN97308	*L. usitatissimum*/O	IND	1	A	3	A	1	A	1	A	2	A	18	D
T.126	CN97312	*L. mediterraneum*/O	IND	1	A	3	A	20	A	1	A	2	A	1	A

Accession/Description	Accession number	Species/type[2]	Origin[3]	***sad* allele and isoform**				***fad2* allele and isoform**				***fad3* allele and isoform**			
				sad1		***sad2***		***fad2a***		***fad2b***		***fad3a***		***fad3b***	
Clli-2528	CN97321	*L. usitatissimum*/O	ROM	1	A	1	A	20	A	1	A	1	A	1	A
Mocoreta	CN97334	*L. usitatissimum/O*	ARG	2	A	3	A	1	A	1	A	2	A	1	A
H723 F3-6-3-3-4-2-2	CN97341	/UN	ARG	5	C	2	B	1	A	5	B	2	A	1	A
de metcha 1-3-3 Vilm	CN97350	*L. usitatissimum*/O	FRA	2	A	1	A	1	A	1	A	2	A	1	A
de metcha 1-3-6 Vilm	CN97351	*L. elongatum*/F	FRA	1	A	1	A	4	A	1	A	15	F	5	D
Texas S. 4-6 Walsh x New Golden	CN97366	*L. usitatissimum*/O	USA	1	A	1	A	3	A	1	A	2	A	12	G
Reserve (N. Dak. Res. 155)	CN97377	*L. usitatissimum*/O	USA	1	A	3	A	8	A	1	A	2	A	5	D
Novelty	CN97392	*L. usitatissimum*/O	CAN	1	A	1	A	1	A	1	A	1	A	5	D
Sel. C.I. 21-2 Jalaun	CN97393	*L. usitatissimum*/O	USA	1	A	3	A	15	B	1	A	2	A	1	A
Res. x Hoshangabad (C.I. 19 x C.I.	CN97396	*L. usitatissimum*/O	USA	1	A	5	A	4	A	1	A	5	A	4	D
Sel. C.I. 19-47 Pale Blue	CN97397	*L. usitatissimum*/UN	USA	1	A	2	B	4	A	1	A	1	A	5	D
No. Dak. No. 40,013	CN97402	/UN	USA	2	A	2	B	14	A	1	A	1	A	7	A
Linota	CN97403	*L. usitatissimum*/O	USA	1	A	3	A	3	A	1	A	1	A	1	A
Buda Sel.	CN97404	*L. usitatissimum*/O	USA	1	A	2	B	2	A	1	A	1	A	7	A
Buda Sel.B	CN97404B	/O	USA	1	A	2	B	14	A	1	A	1	A	7	A
No.Dak.Res. No.52	CN97406	/UN	USA	1	A	1	A	9	C	1	A	1	A	1	A
Rio (Long 79)	CN97407	*L. usitatissimum*/O	USA	2	A	1	A	1	A	1	A	1	A	1	A
Tammes #3 White Involute	CN97424	*L. elongatum*/F	NLD	1	A	1	A	1	A	2	D	1	A	13	G
N.D. Nur. No. 1740 (G.36 a/21)	CN97430	*L. usitatissimum*/O	DEU	1	A	1	A	9	C	1	A	7	A	1	A
TMP 2998-9	CN97430B	*L. usitatissimum* /O	DEU	1	A	1	A	9	C	1	A	7	A	1	A
CDC Bethune	CDC Bethune	*L. usitatissimum* /O	CAN	1	A	2	B	1	A	1	A	8	A	1	A
FP2214	FP2214	*L. usitatissimum* /O	CAN	1	A	1	A	9	C	1	A	1	A	1	A
SP2047*	SP2047*	*L. usitatissimum* /O	CAN	1	A	3	A	10	C	1	A	6	D	16	C
FP2270	FP2270	*L. usitatissimum* /O	CAN	1	A	3	A	1	A	1	A	3	A	1	A
UGG5-5	UGG5-5	*L. usitatissimum* /O	CAN	1	A	3	A	20	A	1	A	3	A	1	A
Hanley	Hanley	*L. usitatissimum* /O	CAN	1	A	2	B	7	C	1	A	1	A	1	A
E1747*	E1747*	*L. usitatissimum* /O	CAN	1	A	3	A	7	C	1	A	4	E	17	B
Lirina	Lirina	*L. usitatissimum* /O	CAN	2	A	4	A	7	C	1	A	3	A	1	A
Atlas	Atlas	*L. usitatissimum* /F	SWE	1	A	1	A	2	A	1	A	1	A	3	D
M5791	M5791	*L. usitatissimum* /O	CAN	1	A	3	A	2	A	1	A	3	A	2	A
Crepitam Tabor	Crepitam Tabor	*L. usitatissimum* /F	HUN	3	A	1	A	1	A	1	A	2	A	5	D
Prairie Blue	Prairie Blue	*L. usitatissimum* /O	CAN	1	A	3	A	6	C	1	A	3	A	1	A
Viking(European)	Viking (European)	*L. usitatissimum* /F	EU	1	A	2	B	1	A	1	A	2	A	1	A
Prairie Grande	Prairie Grande	*L. usitatissimum* /O	CAN	1	A	2	B	7	C	1	A	1	A	1	A
Double Low*	Double Low*	*L. usitatissimum* /O	CAN	1	A	1	A	1	A	1	A	6	D	4	D
Prairie Thunder	Prairie Thunder	*L. usitatissimum* /O	CAN	1	A	1	A	1	A	1	A	1	A	1	A
UGG102-2	UGG102-2	*L. usitatissimum* /O	CAN	1	A	3	A	20	A	1	A	14	C	4	D
S95407*	S95407*	*L. usitatissimum* /O	CAN	3	A	3	A	1	A	1	A	4	E	8	F
UGG146-1*	UGG146-1*	*L. usitatissimum* /O	CAN	1	A	1	A	20	A	1	A	6	D	4	D

**Accession/Description**	**Accession number**	**Species/type**[2]	**Origin**[3]	***sad* allele and isoform**				***fad2* allele and isoform**				***fad3* allele and isoform**			
				sad1		***sad2***		***fad2a***		***fad2b***		***fad3a***		***fad3b***	
YSED18*	YSED18*	*L. usitatissimum* /O	CAN	1	A	2	B	9	C	1	A	4	E	17	B
G-1186-94	G-1186-94	*L. usitatissimum* /F	GER	1	A	1	A	1	A	1	A	3	A	5	D
CDCMons	CDCMons	*L. usitatissimum* /O	CAN	1	A	3	A	14	A	1	A	1	A	1	A
M96006*	M96006*	*L. usitatissimum* /O	CAN	1	A	3	A	1	A	1	A	9	E	8	F
Macbeth	Macbeth	*L. usitatissimum* /O	CAN	1	A	1	A	16	A	1	A	1	A	1	A

[1] Canadian National accession number at the Plant Gene Resources of Canada (PGRC), Saskatoon, SK, Canada
[2] UN = unknown, O = oil seed/linseed type, F = fiber type
[3] Country code
*Induced mutants or derived from induced mutant line
