# Supplementary material for: Genetic variation of six desaturase genes in flax and their impact on fatty acid composition
Source: Theor Appl Genet. 2013 Aug 9;126(10):2627–41. doi: 10.1007/s00122-013-2161-2 (PMC3782649; doi:10.1007/s00122-013-2161-2)
Supplement: Supplementary file 2 — Supplementary material 2 (PDF 113 kb) [file 122_2013_2161_MOESM2_ESM.pdf]

Table S2. Description of primers used for PCR amplification of *sad1, sad2, fad2a, fad2b, fad3a* and *fad3b* and for sequencing

Gene	GeneBank Accession Number	Primer name	Sequence (5' to 3')	Tm (°C)
sad1	AJ006957.1	SAD1-F212N17	AAGCTGCTGCCAAGATTCAT	60.0
		SAD1-R212N17	TACAAGTGGCGAGTGCTGAC	60.1
		SAD1-F49	CTCAACAACTTCTCCTCCAG	55
		SAD1-R357	CTGTGCAAACACTGACAATC	55.1
		SAD1-F626	AGGTCCTTGGAAGAATGACT	55.3
		SAD1-R958	CTGAGCTCCTTCACTTGCTC	57.9
		SAD1-F1213	TTCAGTATCTCATCGGCTCT	55
		SAD1-R1525	TCACGTGTATCCTTTCACAA	55
		SAD1-F1657	TAGGTTTGCCCGTTAGTAAG	54.8
		SAD1-R1938	GAACAAAGTCCAAAAAGCAC	55
		SAD1-F2188	GGACATGATGAGGAAGAAGA	55.1
		SAD1-R2505	AATTCTCTGCTGAAGATCCA	55
sad2	AJ006958.1	SAD2-F6M22	TACAAGTGGCGAGTGCTGAC	60.1
		SAD2-R6M22	GGTTGAATCGCTGAATTGGT	59.9
		SAD2-F49	CTCAACAACTTCTCCTCCAG	55
		SAD2-R339	AATCCAGACTCGTGAAACAC	55.1
		SAD2-F653	TGACATGATTAAGGCGTAGT	53.3
		SAD2-R1000	CCAGCACAACAAAATAGTCA	54.8
		SAD2-F1211	TTCAGTATCTCATCGGCTCT	55
		SAD2-R1559	TTAATTGCACACGACATCAC	55
		SAD2-F1805	TTGTTTGTATCGCTGTATCG	54.9
		SAD2-R2143	ACGATCTTGGTGTATGCTGT	55.6
		SAD2-F2192	GGACATGATGAGGAAGAAGA	55.1
		SAD2-R2509	AATTCTCTGCTGAAGATCCA	55
fad2a	EU660502.1	FAD2A-F139G15	TTGATGTAGGGGAAGAATCCA	59.4
		FAD2A-R139G15	TTCGAAGACCCTCACAGCTT	60.0
		F1143-139G15	ATTCGTCCCTCCTTGTTCCT	59.9
		R1382-139G15	CGAACCGGTCATATGGTCTC	60.3
		FAD2A-F1453	AGGGATATTCACCGTGTGCT	59.4
		FAD2A-R2229	GTTTTGCGATTGCATCATTG	60.1
fad2b	EU660501.1	FAD2B-F25C5	GAACGAAAGCCAAATCCAAA	60.1
		FAD2B-R25C5	GGGAGGGCATTATCCTTGTT	60.2
		F26443-25C5	GCCAGCATCGGAGAAGAATA	60.3
		R26667-25C5	TTTGTCCCCAAGCAGAAATC	60.1
fad3a	HM991829.1	F1499-395P20	CCGTTGCCTAAACTGAAACC	59.6
		R5039-395P20	AGCCTGCAGCATACATCAGA	59.6
		F2063-395P20	GATTGCTCAAGGAACCATGT	57.6
		R2194-395P20	GCAACAGCCCAGATAAAAAG	57.5
		F2604-395P20	CTCTCCCAATGTTTGCGTAT	57.7
		R2730-395P20	TTACCCACCCGAAACATATC	57.3

Gene	GeneBank Accession Number	Primer name	Sequence (5' to 3')	Tm (°C)
		F3177-395P20	TATGGTTTTACCCCAATGGA	57.7
		R3336-395P20	AAGTACATCCATCCACGACA	56.3
		F3781-395P20	CGCATTTCAACCCATACAG	57.6
		R3839-395P20	AATCATGACCGATGTCCTCT	56.9
		F4257-395P20	AGCTGGTCCCTTGAATTTCT	57.9
		R4377-395P20	GGAAAACTGCTCAGGACATT	56.8
fad3b	HM991834.1	FAD3B-F356B4	CCATCCACTTGGCATCCTAC	60.3
		FAD3B-R356B4	AACGCAACCAGAGAGCAGTT	60.1
		F16473-27L18	TGAACAATGTGATGGGACAT	57
		F16946-27L18	GACTCGGGTCGATTTATTTC	56.2
		F17485-27L18	CATTCTCGACCGAAAAGATT	56.9
		F18095-27L18	TCAAATGCCACACTATCACC	56.9
		F18661-27L18	CCCTTATATTCTCAGCCGTTA	56.2
		R16437-27L18	CATGCAACAACCCAGATAAA	57.1
		R16980-27L18	GGTAAGTACCCACCCGAAAT	57.8
		R17583-27L18	GCGGAAAACGACACCTATAC	57.2
		R18114-27L18	GGTGATAGTGTGGCATTTGA	56.9
		R18741-27L18	ATGGATCCGATCTACAAAGC	56.7
