# Supplementary material for: Genetic variation of six desaturase genes in flax and their impact on fatty acid composition
Source: Theor Appl Genet. 2013 Aug 9;126(10):2627–41. doi: 10.1007/s00122-013-2161-2 (PMC3782649; doi:10.1007/s00122-013-2161-2)
Supplement: Supplementary file 3 — Supplementary material 3 (PDF 315 kb) [file 122_2013_2161_MOESM3_ESM.pdf]

Table S3. Fatty acid composition, oil content, iodine value and stearate, oleic and linoleic desaturation proportion of 120 flax accessions averaged from two locations (MB and SK) over three years (2009, 2010 and 2011)

Accession description	Accession no.	Palmitic acid (PAL)		Stearic acid (STE)		Oleic acid (OLE)		Linoleic acid (LIO)		Linolenic acid (LIN)		Oil (OIL)		Iodine value (IOD)[3]		Stearate desaturation proportion (SDP)[4]		Oleic desaturation proportion (ODP)[5]		Linoleic desaturation proportion (LDP)[6]	
		Percent[1]	SE[2]	Percent	SE	Percent	SE	Percent	SE	Percent	SE	Percent	SE	IOD	SE	Proportion	SE	Proportion	SE	Proportion	SE
AC Watson	CN18973	4.85	0.14	4.00	0.23	19.40	1.75	15.05	0.26	56.72	1.69	44.22	0.78	191.12	3.34	0.96	0.00	0.79	0.02	0.79	0.00
Flanders	CN18979	5.01	0.07	4.33	0.34	19.68	1.97	13.79	0.49	57.15	1.87	44.48	1.01	190.32	3.90	0.95	0.00	0.78	0.02	0.81	0.00
Somme	CN18980	5.60	0.21	3.23	0.18	19.06	1.67	14.22	0.24	57.96	1.38	43.32	0.91	192.62	2.58	0.97	0.00	0.79	0.02	0.80	0.00
CDC Valour	CN18981	5.19	0.10	3.18	0.17	23.05	1.97	13.06	0.08	55.62	2.10	43.05	0.91	187.93	3.83	0.97	0.00	0.75	0.02	0.81	0.01
Evelin	CN18982	4.76	0.07	4.87	0.37	19.22	1.96	18.28	0.57	52.82	1.84	39.57	0.57	186.36	3.87	0.95	0.00	0.79	0.02	0.74	0.01
Laura	CN18983	5.17	0.08	5.75	0.40	19.74	1.86	14.81	0.41	54.53	1.93	38.32	0.61	185.29	4.16	0.94	0.00	0.78	0.02	0.79	0.00
Hermes	CN18986	5.03	0.03	4.06	0.27	19.92	1.86	17.45	0.31	53.54	2.00	40.55	0.34	187.40	3.86	0.96	0.00	0.78	0.02	0.75	0.01
Viking	CN18987	4.94	0.02	3.84	0.20	18.96	1.89	16.82	0.43	55.36	1.93	38.75	0.32	190.26	3.69	0.96	0.00	0.79	0.02	0.77	0.01
Ariane	CN18988	5.41	0.08	3.35	0.24	17.78	1.89	16.75	0.27	56.70	1.86	38.98	0.33	192.63	3.55	0.96	0.00	0.80	0.02	0.77	0.00
Atalante	CN18989	6.27	0.17	4.50	0.29	16.67	1.25	12.97	0.19	59.49	1.29	42.07	0.94	192.43	2.64	0.95	0.00	0.81	0.01	0.82	0.00
Nike	CN18991	5.24	0.10	5.42	0.38	22.53	1.77	16.93	0.70	49.76	2.24	40.23	0.81	178.86	4.18	0.94	0.00	0.75	0.02	0.75	0.01
Linda	CN18993	6.14	0.08	5.40	0.27	22.09	1.98	12.68	0.33	53.63	1.94	43.03	0.49	181.24	3.94	0.94	0.00	0.75	0.02	0.81	0.00
Verne	CN18994	5.35	0.08	3.76	0.18	20.12	1.90	15.42	0.15	55.21	1.99	43.23	0.60	188.45	3.74	0.96	0.00	0.78	0.02	0.78	0.01
Raisa	CN18997	5.31	0.06	3.54	0.12	22.95	1.81	16.02	0.36	52.30	1.66	41.11	0.29	184.29	3.20	0.96	0.00	0.75	0.02	0.77	0.00
Escalina	CN18998	5.19	0.06	5.34	0.39	17.73	1.68	16.98	0.52	54.71	1.73	39.27	0.50	187.77	3.71	0.94	0.00	0.80	0.02	0.76	0.00
Marina	CN19001	5.13	0.10	5.23	0.32	19.55	1.70	14.91	0.46	55.05	1.61	39.55	0.69	186.65	3.51	0.94	0.00	0.78	0.02	0.79	0.00
AC McDuff	CN19003	6.01	0.12	4.11	0.26	18.41	1.51	16.67	0.41	54.75	1.49	46.11	0.93	187.93	3.03	0.96	0.00	0.79	0.02	0.77	0.00
AC Emerson	CN19004	5.63	0.11	2.65	0.13	17.43	1.62	14.77	0.14	59.55	1.60	43.68	0.72	196.35	2.98	0.97	0.00	0.81	0.02	0.80	0.00
AC Linora	CN19005	5.73	0.13	2.85	0.15	18.25	1.76	16.60	0.35	56.55	1.77	44.63	0.91	192.39	3.31	0.97	0.00	0.80	0.02	0.77	0.01
no name	CN19007	5.68	0.22	4.54	0.34	22.18	2.05	13.84	0.45	53.75	2.07	41.76	1.40	183.67	4.18	0.95	0.00	0.75	0.02	0.79	0.00
CDC Normandy	CN19017	5.42	0.16	2.99	0.15	23.66	2.71	12.47	0.19	55.36	2.61	42.85	0.86	186.76	4.83	0.97	0.00	0.74	0.03	0.82	0.00
Ottawa 829-C	CN19157	4.92	0.05	2.80	0.08	15.12	1.00	14.46	0.33	62.81	0.67	40.53	0.40	202.35	1.44	0.97	0.00	0.84	0.01	0.81	0.00
Ottawa 770B	CN19158	5.65	0.06	4.23	0.26	15.65	1.28	14.68	0.24	59.99	1.34	42.58	0.45	195.81	2.61	0.96	0.00	0.83	0.01	0.80	0.00
Diadem	CN19159	5.52	0.06	4.19	0.21	21.36	1.67	14.91	0.35	54.03	1.51	43.69	0.59	185.53	3.03	0.96	0.00	0.76	0.02	0.78	0.00
Bolley Golden	CN19160	6.12	0.15	3.78	0.25	17.10	1.71	13.06	0.30	59.85	1.94	34.10	8.65	193.88	3.76	0.96	0.00	0.81	0.02	0.82	0.01
Kirovogradskij 71	CN30860	5.81	0.14	3.92	0.27	21.01	1.87	14.56	0.27	54.58	2.05	43.68	0.66	186.06	4.11	0.96	0.00	0.77	0.02	0.79	0.00
Kubanskij	CN30861	5.35	0.11	3.23	0.12	18.90	2.28	13.10	0.26	59.28	2.21	45.49	1.16	194.01	4.23	0.97	0.00	0.79	0.03	0.82	0.00
Vniil-17	CN32542	5.49	0.21	2.85	0.19	23.44	2.35	12.23	0.26	55.92	2.18	42.56	0.77	187.63	3.99	0.97	0.00	0.74	0.03	0.82	0.00
Korostenskij 3	CN32546	4.62	0.06	4.33	0.25	20.16	1.84	15.84	0.50	55.10	1.54	40.08	0.48	188.93	3.19	0.95	0.00	0.78	0.02	0.78	0.00
Linott	CN33385	4.99	0.04	2.63	0.11	22.57	1.94	14.21	0.31	55.72	1.79	43.06	0.42	189.79	3.28	0.97	0.00	0.76	0.02	0.80	0.01
Noralta	CN33386	6.35	0.15	3.99	0.28	18.14	1.59	15.26	0.28	56.28	1.70	42.02	0.73	189.25	3.49	0.96	0.00	0.80	0.02	0.79	0.00
Redwood 65	CN33388	6.27	0.19	3.16	0.18	19.19	1.50	16.14	0.28	55.31	1.38	43.62	0.97	189.14	2.59	0.97	0.00	0.79	0.02	0.77	0.00

Accession description	Accession no.	Palmitic acid (PAL)		Stearic acid (STE)		Oleic acid (OLE)		Linoleic acid (LIO)		Linolenic acid (LIN)		Oil (OIL)		Iodine value (IOD)[3]		Stearate desaturation proportion (SDP)[4]		Oleic desaturation proportion (ODP)[5]		Linoleic desaturation proportion (LDP)[6]	
		Percent[1]	SE[2]	Percent	SE	Percent	SE	Percent	SE	Percent	SE	Percent	SE	IOD	SE	Proportion	SE	Proportion	SE	Proportion	SE
Rocket	CN33389	5.95	0.13	4.16	0.30	18.80	1.63	12.38	0.20	58.76	1.91	42.91	0.65	191.32	3.52	0.96	0.00	0.79	0.02	0.83	0.01
Natasja	CN33390	4.87	0.07	5.33	0.49	18.73	1.63	17.47	0.43	53.55	1.79	38.71	0.68	186.45	3.88	0.94	0.01	0.79	0.02	0.75	0.00
Domtar Selection	CN33393	4.73	0.09	4.85	0.33	18.15	1.58	15.68	0.54	56.67	1.57	39.46	0.50	191.01	3.28	0.95	0.00	0.80	0.02	0.78	0.01
Dufferin	CN33397	5.31	0.10	4.34	0.32	19.66	1.90	14.74	0.40	55.88	1.97	43.47	1.14	188.62	4.06	0.95	0.00	0.78	0.02	0.79	0.00
Bison	CN33399	5.62	0.06	3.17	0.12	24.74	2.45	14.44	0.36	52.12	2.26	42.93	0.50	182.64	4.22	0.97	0.00	0.73	0.03	0.78	0.01
Norstar	CN33400	6.24	0.07	2.94	0.17	20.94	1.90	16.56	0.30	53.25	2.13	43.10	0.56	185.99	3.90	0.97	0.00	0.77	0.02	0.76	0.01
Culbert	CN33992	4.48	0.06	3.49	0.16	16.72	1.51	15.40	0.19	59.88	1.64	43.91	0.34	197.69	3.10	0.96	0.00	0.82	0.02	0.80	0.00
Tverca	CN35791	5.03	0.04	3.66	0.20	22.22	1.69	15.24	0.55	53.98	1.46	42.53	0.17	186.70	3.00	0.96	0.00	0.76	0.02	0.78	0.01
McGregor	CN37286	6.08	0.19	4.27	0.27	17.13	1.36	17.39	0.51	55.38	1.07	42.26	0.98	189.73	2.31	0.95	0.00	0.81	0.02	0.76	0.00
Natasja	CN40081	5.03	0.09	5.38	0.49	19.60	1.99	17.14	0.41	52.91	2.08	38.45	0.76	184.96	4.33	0.94	0.01	0.78	0.02	0.75	0.00
Norlin	CN52732	5.32	0.13	3.05	0.15	24.27	2.11	12.38	0.19	54.89	1.92	43.11	0.74	185.90	3.53	0.97	0.00	0.73	0.02	0.82	0.00
Clli-642	CN96845	5.73	0.11	3.92	0.17	14.68	1.40	12.96	0.46	62.85	1.62	41.60	0.77	199.50	2.72	0.96	0.00	0.84	0.02	0.83	0.01
Clli-643	CN96846	5.74	0.09	3.43	0.17	19.98	1.61	11.71	0.37	59.23	1.39	43.38	0.63	192.40	2.84	0.96	0.00	0.78	0.02	0.84	0.00
Clli-1407	CN96911	6.44	0.21	4.44	0.23	22.02	1.70	13.76	0.32	53.39	1.64	43.37	0.97	182.44	3.36	0.95	0.00	0.75	0.02	0.79	0.00
Clli-1455	CN96958	6.00	0.21	4.63	0.40	25.22	1.98	9.22	1.07	54.95	1.93	46.28	1.70	181.40	4.37	0.95	0.00	0.72	0.02	0.86	0.01
Clli-1458	CN96962	6.14	0.14	4.31	0.33	23.36	1.81	12.03	0.54	54.24	1.60	42.87	0.85	182.81	3.52	0.95	0.00	0.74	0.02	0.82	0.00
Clli-1470	CN96974	5.94	0.11	5.23	0.23	40.06	2.74	5.73	0.61	43.02	2.40	43.56	0.89	156.93	4.98	0.94	0.00	0.55	0.03	0.88	0.00
Clli-1499	CN96988	5.80	0.17	4.11	0.24	15.32	1.72	12.72	0.22	62.06	2.02	42.64	0.58	197.56	3.88	0.96	0.00	0.83	0.02	0.83	0.00
Clli-1502	CN96991	6.01	0.22	4.73	0.16	20.79	2.32	12.29	0.51	56.13	2.36	38.52	0.99	185.99	3.97	0.95	0.00	0.77	0.03	0.82	0.01
Clli-1503	CN96992	6.31	0.18	5.61	0.42	19.64	1.73	12.87	0.85	55.57	1.50	36.71	2.01	184.55	3.87	0.94	0.00	0.78	0.02	0.81	0.01
Clli-1519	CN97004	5.80	0.16	4.88	0.25	22.10	0.83	13.81	0.28	53.53	0.53	39.41	1.45	182.96	0.88	0.95	0.00	0.75	0.01	0.79	0.00
Clli-1924	CN97050	5.64	0.12	5.21	0.25	24.60	1.97	9.97	0.55	54.54	1.73	43.11	0.57	181.09	3.76	0.94	0.00	0.72	0.02	0.85	0.00
Clli-1930	CN97056	5.83	0.14	5.26	0.28	26.59	2.17	9.57	0.51	52.71	2.00	44.13	0.97	177.33	4.23	0.94	0.00	0.70	0.03	0.85	0.00
Clli-1938	CN97064	5.72	0.15	5.49	0.33	27.00	1.89	9.60	0.43	52.22	1.93	44.42	1.01	176.45	4.12	0.94	0.00	0.70	0.02	0.84	0.00
Clli-1946	CN97072	5.97	0.17	10.47	0.84	21.39	1.53	11.00	0.44	51.20	1.99	43.54	1.23	171.38	4.64	0.89	0.01	0.74	0.02	0.82	0.00
Clli-1957	CN97083	5.45	0.11	5.17	0.32	26.34	2.27	8.64	0.48	54.59	2.12	43.93	0.71	180.42	4.39	0.95	0.00	0.71	0.03	0.86	0.00
Clli-1991	CN97092	6.51	0.24	5.10	0.33	21.91	1.81	14.12	0.24	52.38	1.82	45.76	1.27	180.33	3.56	0.95	0.00	0.75	0.02	0.79	0.00
Clli-1995	CN97096	6.08	0.13	4.44	0.25	19.90	1.97	13.66	0.61	55.92	1.95	46.16	1.33	187.05	3.88	0.95	0.00	0.78	0.02	0.80	0.01
Clli-2002	CN97103	5.73	0.16	4.18	0.32	22.81	1.81	15.26	0.44	52.01	1.80	43.03	0.62	182.10	3.64	0.96	0.00	0.75	0.02	0.77	0.01
Clli-2028	CN97129	5.92	0.16	4.09	0.29	21.84	2.42	12.37	0.46	55.62	2.82	43.28	0.74	185.70	5.22	0.96	0.00	0.76	0.03	0.82	0.01
Clli-2028B	CN97129B	6.16	0.16	4.00	0.23	19.60	1.91	11.97	0.43	58.40	1.84	42.81	0.92	190.36	3.63	0.96	0.00	0.78	0.02	0.83	0.00
Clli-2038	CN97139	5.72	0.11	5.78	0.38	22.22	1.66	12.09	0.35	54.16	1.78	43.38	0.81	181.72	3.75	0.94	0.00	0.75	0.02	0.82	0.00
Clli-2046	CN97147	6.03	0.07	4.16	0.20	20.86	1.70	13.37	0.31	55.60	1.60	43.74	0.47	186.55	3.25	0.96	0.00	0.77	0.02	0.81	0.00

Accession description	Accession no.	Palmitic acid (PAL)		Stearic acid (STE)		Oleic acid (OLE)		Linoleic acid (LIO)		Linolenic acid (LIN)		Oil (OIL)		Iodine value (IOD)[3]		Stearate desaturation proportion (SDP)[4]		Oleic desaturation proportion (ODP)[5]		Linoleic desaturation proportion (LDP)[6]	
		Percent[1]	SE[2]	Percent	SE	Percent	SE	Percent	SE	Percent	SE	Percent	SE	IOD	SE	Proportion	SE	Proportion	SE	Proportion	SE
Clli-2052	CN97153	6.31	0.17	4.60	0.33	20.56	1.69	13.27	0.17	55.22	1.94	43.70	0.96	185.11	3.83	0.95	0.00	0.77	0.02	0.81	0.00
Horal	CN97176	6.00	0.07	5.57	0.26	30.08	2.39	9.44	0.41	48.93	2.38	43.58	0.75	170.22	4.69	0.94	0.00	0.66	0.03	0.84	0.01
Sorth Behbehan	CN97180	5.19	0.15	4.10	0.14	19.08	1.35	13.94	0.31	57.71	1.15	43.70	1.07	191.52	2.27	0.96	0.00	0.79	0.02	0.81	0.00
noname	CN97214	6.07	0.15	3.29	0.23	17.52	1.46	12.07	0.29	61.02	1.58	43.01	1.23	195.58	3.29	0.96	0.00	0.81	0.02	0.83	0.00
No. 1048	CN97238	6.03	0.15	4.95	0.27	25.38	2.05	13.87	0.44	49.81	1.97	42.34	1.11	176.14	4.12	0.95	0.00	0.71	0.02	0.78	0.00
Lina Deta	CN97287	5.89	0.09	4.06	0.28	23.77	2.24	12.53	0.38	53.73	2.13	42.85	1.06	182.69	4.30	0.96	0.00	0.74	0.03	0.81	0.00
Raja	CN97300	5.41	0.14	4.32	0.21	21.12	1.47	15.45	0.67	53.76	1.56	41.15	0.57	185.54	3.09	0.95	0.00	0.77	0.02	0.78	0.01
N.P. (R.R.) 9	CN97306	5.95	0.15	6.00	0.28	23.50	1.53	12.56	0.28	51.97	1.69	46.29	0.95	177.93	3.52	0.94	0.00	0.73	0.02	0.81	0.00
N.P. (R.R.) 37	CN97307	5.98	0.30	5.34	0.38	23.56	2.30	11.11	0.69	54.11	2.23	44.33	1.08	181.05	4.93	0.94	0.00	0.73	0.03	0.83	0.00
N.P. (R.R.) 38	CN97308	5.67	0.26	5.08	0.86	24.72	1.41	10.39	0.67	54.17	1.58	44.70	1.97	180.96	3.45	0.95	0.01	0.72	0.02	0.84	0.01
T.126	CN97312	6.24	0.22	7.77	0.51	28.98	2.14	11.45	0.30	45.55	2.42	42.23	0.83	163.91	5.04	0.92	0.01	0.66	0.03	0.80	0.00
Clli-2528	CN97321	5.86	0.10	4.44	0.24	21.59	1.90	13.40	0.34	54.73	1.80	43.15	0.92	184.96	3.63	0.95	0.00	0.76	0.02	0.80	0.00
Mocoreta	CN97334	5.40	0.17	4.39	0.21	21.76	1.95	14.86	0.40	53.67	1.68	44.18	0.91	184.83	3.35	0.95	0.00	0.76	0.02	0.78	0.00
H723 F3-6-3-3-4-2-2	CN97341	5.40	0.09	6.62	0.35	18.59	1.03	13.12	0.45	56.28	1.01	43.44	0.31	185.93	2.44	0.93	0.00	0.79	0.01	0.81	0.00
de metcha 1-3-3 Vilm	CN97350	5.81	0.09	3.73	0.17	19.11	1.49	14.81	0.41	56.40	1.46	41.51	0.81	189.63	2.93	0.96	0.00	0.79	0.02	0.79	0.00
de metcha 1-3-6 Vilm	CN97351	5.71	0.16	5.61	0.45	24.18	2.33	11.76	0.60	52.70	2.25	39.68	0.73	179.03	4.69	0.94	0.00	0.73	0.03	0.82	0.01
Texas S. 4-6 Walsh x New Golden	CN97366	5.79	0.10	3.02	0.18	19.22	1.76	14.69	0.25	57.29	1.75	44.84	0.79	191.83	3.24	0.97	0.00	0.79	0.02	0.80	0.00
Reserve (N. Dak. Res. 155)	CN97377	5.56	0.07	3.47	0.17	18.15	1.65	13.24	0.32	59.59	1.53	42.71	0.60	194.42	3.10	0.96	0.00	0.80	0.02	0.82	0.00
Novelty	CN97392	5.13	0.04	3.52	0.11	18.65	1.83	15.95	0.47	56.62	1.51	41.94	0.50	191.77	3.16	0.96	0.00	0.80	0.02	0.78	0.00
Sel. C.I. 21-2 Jalaun	CN97393	5.84	0.08	3.93	0.18	14.88	1.23	12.80	0.33	62.65	1.41	40.49	0.60	198.87	2.49	0.96	0.00	0.84	0.01	0.83	0.01
Res. x Hoshangabad (C.I. 19 x C.I.	CN97396	5.84	0.06	3.94	0.11	24.70	1.93	13.38	0.24	52.14	1.82	46.28	0.77	180.81	3.48	0.96	0.00	0.73	0.02	0.80	0.00
Sel. C.I. 19-47 Pale Blue	CN97397	5.85	0.05	3.10	0.15	17.47	1.41	16.68	0.44	56.93	1.14	42.45	0.40	192.85	2.47	0.97	0.00	0.81	0.02	0.77	0.00
No. Dak. No. 40,013	CN97402	5.65	0.05	2.32	0.07	21.09	1.88	13.38	0.49	57.58	1.54	41.09	0.37	191.93	3.09	0.98	0.00	0.77	0.02	0.81	0.00
Linota	CN97403	4.69	0.05	2.84	0.06	18.47	1.37	17.34	0.50	56.59	1.17	39.83	0.22	193.95	2.32	0.97	0.00	0.80	0.01	0.77	0.01
Buda Sel.	CN97404	5.67	0.13	2.65	0.24	22.84	3.11	13.42	0.33	55.42	2.91	42.07	1.18	187.87	5.44	0.97	0.00	0.75	0.03	0.80	0.01
Buda Sel.B	CN97404B	5.65	0.06	2.41	0.14	23.56	2.58	13.57	0.42	54.83	2.33	42.03	0.83	187.21	4.49	0.97	0.00	0.74	0.03	0.80	0.00
No.Dak.Res. No.52	CN97406	4.74	0.09	3.02	0.12	17.75	1.52	14.99	0.14	59.38	1.48	42.06	0.64	196.55	2.75	0.97	0.00	0.81	0.02	0.80	0.00
Rio (Long 79)	CN97407	6.03	0.22	4.76	0.29	20.99	1.61	13.96	0.52	54.28	1.32	42.69	1.01	184.22	2.92	0.95	0.00	0.76	0.02	0.80	0.00
Tammes #3 White Involute	CN97424	5.00	0.06	3.36	0.12	16.01	1.23	15.50	0.26	60.23	1.14	40.84	0.19	198.17	2.20	0.96	0.00	0.83	0.01	0.80	0.00
N.D. Nur. No. 1740 (G.36 a/21)	CN97430	8.62	0.88	4.61	0.24	20.07	1.28	12.25	0.74	54.39	1.34	44.71	0.88	180.75	3.52	0.95	0.00	0.77	0.02	0.82	0.01
TMP 2998-9	CN97430B	8.51	0.66	4.74	0.25	20.43	1.78	11.40	0.50	54.85	1.93	44.03	0.93	180.80	3.31	0.95	0.00	0.76	0.02	0.83	0.01

Accession description	Accession no.	Palmitic acid (PAL)		Stearic acid (STE)		Oleic acid (OLE)		Linoleic acid (LIO)		Linolenic acid (LIN)		Oil (OIL)		Iodine value (IOD)[3]		Stearate desaturation proportion (SDP)[4]		Oleic desaturation proportion (ODP)[5]		Linoleic desaturation proportion (LDP)[6]	
		Percent[1]	SE[2]	Percent	SE	Percent	SE	Percent	SE	Percent	SE	Percent	SE	IOD	SE	Proportion	SE	Proportion	SE	Proportion	SE
CDC Bethune	CDCBethune	5.14	0.13	3.80	0.25	21.29	1.96	14.98	0.36	54.79	1.86	44.40	0.75	187.59	3.55	0.96	0.00	0.77	0.02	0.78	0.00
FP2214	FP2214	4.96	0.13	3.90	0.25	18.36	2.69	14.74	0.69	57.82	2.34	46.45	0.98	192.57	4.81	0.96	0.00	0.80	0.03	0.80	0.00
SP2047*	SP2047	5.94	0.32	3.71	0.20	17.08	1.76	55.49	12.77	17.77	11.49	48.46	1.19	157.28	9.27	0.96	0.00	0.81	0.02	0.25	0.17
FP2270	FP2270	5.06	0.10	5.47	0.55	17.69	2.35	12.27	0.33	59.60	2.47	42.71	1.08	192.39	5.00	0.94	0.01	0.80	0.03	0.83	0.00
UGG5-5	UGG5-5	4.00	0.10	2.48	0.14	12.96	1.80	11.17	0.32	69.31	1.71	44.54	0.91	211.79	3.42	0.97	0.00	0.86	0.02	0.86	0.00
Hanley	Hanley	5.74	0.10	2.83	0.12	16.52	1.47	17.16	0.30	57.82	1.42	43.04	0.91	195.18	2.60	0.97	0.00	0.82	0.02	0.77	0.00
E1747*	E1747	6.10	0.21	4.08	0.38	16.21	1.68	52.23	3.96	21.38	5.66	41.53	1.24	160.33	6.87	0.96	0.00	0.82	0.02	0.29	0.07
Lirina	Lirina	5.39	0.08	3.78	0.28	19.63	2.75	13.77	0.30	57.36	3.26	48.61	1.32	190.79	5.78	0.96	0.00	0.78	0.03	0.81	0.01
Atlas	Atlas	4.92	0.10	3.85	0.22	20.00	2.88	14.18	0.43	56.97	2.75	42.25	0.45	190.79	5.42	0.96	0.00	0.78	0.03	0.80	0.00
M5791	M5791	4.40	0.06	2.44	0.12	11.41	1.44	9.89	0.28	71.85	1.43	43.73	0.86	214.90	2.69	0.97	0.00	0.88	0.02	0.88	0.00
Crepitam Tabor	Crepitam Tal	5.40	0.10	2.77	0.11	19.93	2.72	14.45	0.33	57.49	2.44	44.47	0.61	192.56	4.58	0.97	0.00	0.78	0.03	0.80	0.00
Prairie Blue	Prairie Blue	5.02	0.10	4.24	0.31	19.29	1.91	13.30	0.40	58.20	1.91	45.01	0.98	191.87	3.74	0.96	0.00	0.79	0.02	0.81	0.00
Viking(European)	Viking	4.96	0.06	3.80	0.26	18.34	2.49	16.38	0.51	56.65	2.18	38.83	0.46	192.35	4.44	0.96	0.00	0.80	0.03	0.78	0.00
Prairie Grande	Prairie Granc	4.69	0.07	3.92	0.21	19.89	1.61	14.55	0.28	56.90	1.57	45.23	0.52	191.15	3.16	0.96	0.00	0.78	0.02	0.80	0.00
Double Low*	Double Low	5.79	0.19	4.43	0.35	19.50	2.46	25.13	0.83	45.23	2.79	45.55	1.51	178.61	4.87	0.95	0.00	0.78	0.03	0.64	0.02
Prairie Thunder	Prairie Thunc	5.25	0.21	4.09	0.27	18.04	1.61	15.37	0.99	57.25	1.08	44.13	0.87	191.89	2.35	0.96	0.00	0.80	0.02	0.79	0.01
UGG102-2*	UGG102-2	6.18	0.11	4.38	0.35	18.48	2.56	13.80	0.66	56.93	2.32	47.57	0.91	188.72	4.85	0.95	0.00	0.79	0.03	0.80	0.00
S95407*	S95407	6.33	0.15	4.29	0.38	17.62	1.80	67.50	1.68	4.22	0.62	45.57	0.86	143.10	2.95	0.95	0.00	0.80	0.02	0.06	0.01
UGG146-1*	UGG146-1	6.13	0.34	4.72	0.27	19.47	1.89	25.08	0.21	44.59	1.77	48.46	0.42	176.83	3.11	0.95	0.00	0.78	0.02	0.64	0.01
YSED18*	YSED18	6.44	0.24	3.65	0.33	17.34	1.69	64.82	4.02	7.58	2.59	43.56	0.69	147.00	2.11	0.96	0.00	0.81	0.02	0.11	0.04
G-1186-94	G-1186-94	5.95	0.14	3.38	0.17	13.48	1.48	12.12	0.66	65.17	1.58	42.97	0.92	203.07	2.64	0.96	0.00	0.85	0.02	0.84	0.01
CDCMons	CDCMons	5.66	0.25	3.64	0.18	17.58	1.42	15.39	0.35	57.84	1.20	43.68	1.41	193.07	2.23	0.96	0.00	0.81	0.02	0.79	0.00
M96006*	M96006	12.50	1.92	3.63	0.27	15.33	1.62	34.12	3.12	34.02	4.41	42.04	1.21	161.27	7.27	0.96	0.00	0.82	0.02	0.50	0.06
Macbeth	Macbeth	4.93	0.16	3.87	0.20	17.78	1.53	16.11	0.55	57.40	1.31	46.32	0.82	193.34	2.49	0.96	0.00	0.81	0.02	0.78	0.01

[1]Expressed as a percentage of the total fatty acid composition

[2]Standard error

[3]IOD=(0.86×OLE)+(1.732×LIO)+(2.616×LIN); Reference: AOCS Method Cd 1c-85

[4]SDP=(%OLE+%LIO+%LIN)/(%STE+%OLE+%LIO+%LIN); Reference: Zarhoul et al (2006) Mol Breeding 18:241-251

[5]ODP=(%LIO+%LIN)/(%OLE+%LIO+%LIN); Reference: Green (1986b)

[6]LDP=%LIN/(%LIO+%LIN); Reference: Green (1986b)
