# Supplementary material for: Genetic variation of six desaturase genes in flax and their impact on fatty acid composition
Source: Theor Appl Genet. 2013 Aug 9;126(10):2627–41. doi: 10.1007/s00122-013-2161-2 (PMC3782649; doi:10.1007/s00122-013-2161-2)
Supplement: Supplementary file 4 — Supplementary material 4 (PDF 104 kb) [file 122_2013_2161_MOESM4_ESM.pdf]

a

```
sad1-a          ATGGCTCTCAAGCTCAACCCAGTCACCACCTTCCCTTCAACACGCTCCCTCAACAACTTC 60
sad2-a          ATGGCTCTCAAGCTCAACCCAGTCACCACCTTCCCTTCGACCCGCTCCCTCAACAACTTC 60
                ************************************** ** ******************

sad1-a          TCCTCCAGATCTCCTCGCACCTTTCTCATGGCTGCTTCCACTTTCAATTCCACCTCCACC 120
sad2-a          TCCTCCAGATCTCCTCGCACCTTTCTCATGGCTGCTTCCACTTTCAATTCCACTTCCACC 120
                ***************************************************** ******

sad1-a          AAGTAAG----------CATCTCCTCCTCCTCGGAATCTCCGCCGATTTCTTTTAAGCGA 170
sad2-a          AAGTAAGTTCCCGTCACCATCTCCTCTTCCTCGGAATCTCCGCCG-TTTCATTTAAGCGA 179
                *******          ********* ****************** **** *********

sad1-a          TTGATCGTAGATAAATTTGTCGGTTGCTTACCGTTCATCAAAATCTGCACGGTTCGTTTC 230
sad2-a          TTGATCGTAGA-AAATCTGTCGGTTGCTTAGCGTTCATTCAAATCTGCGCGGTTCGTTTC 238
                *********** **** ************* *******  ******** ***********

sad1-a          TTCTTCTGC---------GCCTAGATTGCATTATGTCATTGTTCGTTTTCCGATTTGACT 281
sad2-a          TTTTTCTTTCTTCAGACTGCCTCGTCTGCATTATGTTATTGTTCGTTT-CCGATTTGACT 297
                ** ****           **** *  ********** *********** ***********

sad1-a          GACCGACATAAATCAATTCCTTTGTGTTTCACGATTCTGGGTTTTGCGCTGTAATTGATT 341
sad2-a          AACCTACATAA-TCAATTCCTTTGTGTTTCACGAGTCTGGATTTTGCGCTGTAATTGATT 356
                 *** ****** ********************** ***** *******************

sad1-a          GTCAGTGTTTGCACAGGTTTCCCCTTCTCCTCCTCCGTCCATCAAATGCATGTTATTACC 401
sad2-a          GTCAGCGTTTGCACAGGTTTCCATTTCTCCACCTCCGTCCATCAAATGCATGTTATTACC 416
                ***** ****************  ****** *****************************

sad1-a          ATTTCAATTTCAGTTTCCTTCTCTGAAATATCCGTCTCTGGGAAAATAAGTCTCTGTATC 461
sad2-a          -TACCAATTTCAGCGTCTTTCTCTGGAA-----ATTTCTG----------TCTCTGTATC 460
                 *  *********  ** ******* **      * ****          **********

sad1-a          TACTATCCTATCAGCTTGTTTAGGAGAGGTTCGATATTCGTTTACATAAACCAATTGGCT 521
sad2-a          TACTATCCTATTAGCTTGTTTGAGAGAGGTTCAATATTGGTTTGCATGAACCAAGTGGCT 520
                *********** *********  ********* ***** **** *** ****** *****

sad1-a          TACAGTCCTTGAACGTTCTAAATGTTGGTCGCGGTGATAATAGGTTCTCAAAAGAGGTTT 581
sad2-a          TACAATCCTTCAACGTTCTAAATGTTGGTCGCAGTAACAATAGGTTCTCAAAAGAGGTTT 580
                **** ***** ********************* ** * **********************

sad1-a          GTCTATGTTGTTTGGCAAAATCTTGTTTCTGTGAATCATGTTTAAGGTCCTTGGAAGAAT 641
sad2-a          TTCTATGTTGTTTGGCAAAATCTTGTTTCTGTGAATCATGTT-AAGGTCCTGGGAAGAAT 639
                 ***************************************** ******** ********

sad1-a          GACTAATGAGCTATGACATGATTACGACGTAGTAGTTATTGAACTGCTGATAATTCAATA 701
sad2-a          GATTAATGAGCTATGACATGATTAAGGCGTAGTAGTTATTGAACTGCTGATAATTCAATA 699
                ** ********************* * *********************************

sad1-a          TAGGGGTAACTTTGTTGATTGTTTGGTCACAGGGAGGCTGAGAAGCTAAAGAAGTCACAT 761
sad2-a          TAGGGGTAACTTTGTTGGTTGTTTGGTGACAGGGAGGCTGAGAAGCTAAAGAAGTCACAT 759
                ***************** ********* ********************************

sad1-a          GGACCACCAAAAGAGGTGCATATGCAAGTGACCCATTCCATGCCCCCACAGAAGCTGGAG 821
sad2-a          GGACCACCAAAAGAGGTGCATATGCAAGTGACCCATTCCATGCCCCCACAGAAGCTGGAG 819
                ************************************************************

sad1-a          ATATTTAAGTCTCTGGAAGGTTGGGCTGAGGATGTTCTATTACCGCACCTGAAGCCAGTT 881
sad2-a          ATCTTTAAGTCCCTTGAAGGTTGGGCAGAGGACGTTCTGTTGCCGCACCTGAAGCCGGTT 879
                ** ******** ** *********** ***** ***** ** ************** ***

sad1-a          GAGAAATGCTGGCAGCCACAGGATTTCCTGCCCGAACCTGAGTCGGATGGGTTCGAGGAG 941
sad2-a          GAGAAATGCTGGCAGCCACAAGATTTCCTGCCCGAACCCGAGTCGGATGGGTTCGAGGAG 939
                ******************** ***************** *********************

sad1-a          CAAGTGAAGGAGCTCAGGGCAAGGGCCAAAGAACTGCCCGATGACTATTTTGTTGTGCTG 1001
sad2-a          CAAGTGAAGGAGCTCAGGGCAAGGGCTAAAGAACTCCCCGATGACTATTTTGTTGTGCTG 999
                ************************** ******** ************************
```

```
sad1-a          GTTGGGGATATGATCACCGAAGAAGCTCTGCCGACTTACCAGACAATGCTCAACACCCTT 1061
sad2-a          GTTGGGGATATGATCACCGAAGAAGCTCTACCGACTTACCAGACAATGCTCAACACCCTT 1059
                ***************************** ******************************

sad1-a          GACGGGGTGAGGGACGAGACTGGAGCCAGCCTTACGCCGTGGGCAATCTGGACAAGGGCG 1121
sad2-a          GACGGGGTGAGGGACGAGACTGGAGCCAGCCTTACGCCGTGGGCAATCTGGACAAGGGCG 1119
                ************************************************************

sad1-a          TGGACCGCTGAAGAGAATAGGCACGGTGACCTTCTCAACAAGTATCTATACCTCTCTGGA 1181
sad2-a          TGGACCGCTGAAGAGAATAGGCACGGTGACCTTCTCAACAAGTATCTTTACCTCTCTGGA 1179
                *********************************************** ************

sad1-a          AGGGTGGACATGAGGCAAATTGAAAAGACCATTCAGTATCTCATCGGCTCTGGAATGGTA 1241
sad2-a          AGGGTGGACATGAGGCAAATTGAAAAGACCATTCAGTATCTCATCGGCTCTGGAATGGTA 1239
                ************************************************************

sad1-a          TGTAATCACATACT----------TCATCCTTTTCTATTAATCTTTGCGTGAACAAAATT 1291
sad2-a          TGTACTCACATCCTATCTGCTCCTTTATCCTTTTCCATTAATCTTTGATTGAACAAAATT 1299
                **** ****** **          * ********* ***********  ***********

sad1-a          CACTACACTGGTAGCAGCTGAAACTTTAGATGATTTTTTTTA-CTGCCTAGCTTCTATGA 1350
sad2-a          CAATAAACTGGTAGC---TGAAACTTTAGATGATTTGTTATAACTGCCTAGCTTCTATGA 1356
                ** ** *********   ****************** ** ** *****************

sad1-a          AACAAAACCACGTAAGTCAAATAGGGTTGACAATGAGTTCAAGTGGCAAAATTTTTCTTA 1410
sad2-a          GA--AAACCACTGAAGTCAAATAGGTTTGACAATGGGTTTAAATGGAAAAAGTTTC---A 1411
                 *  *******  ************ ********* *** ** *** **** ***    *

sad1-a          TATACCAACTTCGAACCACTTTATATGACATACCAACTCCTAGTTCGGTTAAAATTCCTC 1470
sad2-a          TATACCATCTTCCATCTATTTTACATGACATACCAACTTCTACTTCGGAGAAAATTCGCC 1471
                ******* **** * * * **** ************** *** *****  *******  *

sad1-a          --------------CGTCGAAGATATAATACTTGG---ATTGGTTAAATGAATTGTGAAA 1513
sad2-a          GTGGATAATCATATTATTGAAGATATAGTACTTAGTAGATTGGTTAGATGAACTGTTAAA 1531
                                * ********* ***** *   ******** ***** *** ***

sad1-a          GGATACACGTGATGTGGTCTGGAATTAATTTGTTTGAATGATCAGTTGGGTTCGGGGCGA 1573
sad2-a          CAATACATGTGATGTCGTGTGCAATTAATTTGTGTAAATGATTAGCTGGGTTCGGGACGA 1591
                  ***** ******* ** ** *********** * ****** ** ********** ***

sad1-a          CAACTGTGAACTGGAACCACCCTAAGTAAATTTTCTTTCTGTCCTACAAATTTGAGGTTC 1633
sad2-a          CAAATGTGAACTGGAACC---CTA-GTAAA-------------CTATGAATT-GAGGTTG 1633
                *** **************   *** *****             ***  **** ******

sad1-a          TCCTTGATCACCTTAGTCCATCTTAGGTTTG----CCCGTTAGTAAGATCTGCATTTAGC 1689
sad2-a          TCCTTCATCACTTTATTCTGTCCTGGGTTTGTTTGCCTGTTTGCAAGATCTGCATGTAGC 1693
                ***** ***** *** **  ** * ******    ** *** * *********** ****

sad1-a          AGTTTGTCCTGGTATTTGATATCACTAGTATCTTTGTTTGATTCCCTAGCATCTCTGAAA 1749
sad2-a          AGTTTGTCCTGGTATTTGCTACCAGTGGTATCTTTGTTTGATTCCCTAGCATCTCTGAAA 1753
                ****************** ** ** * *********************************

sad1-a          CCATCGGAC-AAGTAGGTGGTTTAGGACAAATTTGGTTCATTGCGGCATTTTTTGTTTGT 1808
sad2-a          ACATCGGACCAAGTATCTGGTT-AGGACAAATTTGGTTCATTGCGGCATTTTTTGTTTGT 1812
                 ******** *****  ***** *************************************

sad1-a          ATCGCCGTATCATCTGGAAGAAGCAGACAGTTTTGCAAAGTGGCATCAAGCTCAAGAAAG 1868
sad2-a          ATCGCTGTATCGTCTGGAAGAAGCAGACAGTTTTGCAAAGTGGCATCAAGCTCAAGAAAG 1872
                ***** ***** ************************************************

sad1-a          CAACGGCTAGAAGAAGTTCTACATCTGATGCTTTCCTTTTGTTTCTTTGTGTGCTTTTTG 1928
sad2-a          CAACGGCTAGAAGAAGTTCTACATCTGATGCGTTCCTTTTGTTTCTTTGTGTGCTTTTTG 1932
                ******************************* ****************************

sad1-a          GACTTTGTTCTTTTTTCCTGTAGGATCCAAAAACAGAAAACAACCCCTACCTCGGTTTCA 1988
sad2-a          GACTTTGTTCTTTTTGCCTGTAGGATCCAAAAACAGAAAACAACCCCTACCTCGGTTTCA 1992
                *************** ********************************************

sad1-a          TCTACACCTCATTCCAAGAGAGGGCAACGTTCATCTCCCACGGAAACACAGCCAGACTCG 2048
sad2-a          TCTACACCTCATTCCAAGAGAGGGCAACGTTCATCTCCCACGGAAATACGGCCAGACTCG 2052
                ********************************************** ** **********
```

```
sad1-a          CCAAGGACCATGGGGACATGAAGCTGGCGCAGATCTGCGGGATCATCGCAGCAGACGAGA 2108
sad2-a          CCAAGGACCACGGGGACATGAAGCTGGCGCAGATCTGCGGGATCATCGCAGCAGACGAGA 2112
                ********** *************************************************

sad1-a          AACGGCACGAAACCGCATACACCAAGATCGTCGAGAAGCTCTTCGAGATCGACCCTGACG 2168
sad2-a          AGCGGCACGAAACAGCATACACCAAGATCGTCGAGAAGCTCTTCGAGATCGACCCTGACG 2172
                * *********** **********************************************

sad1-a          GTACAGTGCTGGCACTGGCGGACATGATGAGGAAGAAGATATCGATGCCCGCCCACTTGA 2228
sad2-a          GTACAGTGTTGGCTCTGGCGGACATGATGAGGAAGAAGATATCGATGCCCGCACACTTGA 2232
                ******** **** ************************************** *******

sad1-a          TGTACGATGGAGAAGACGACAACCTCTTCGACAATTACTCGTCAGTCGCTCAACGCATCG 2288
sad2-a          TGTACGATGGAGAAGACGACAACCTCTTCGACAATTACTCGTCGGTCGCTCAACGCATCG 2292
                ******************************************* ****************

sad1-a          GGGTGTATACTGCCAAGGATTATGCCGATATCCTGGAGTTCCTGGTGGGGAGGTGGAAAG 2348
sad2-a          GGGTGTATACTGCCAAGGATTATGCTGATATCCTGGAGTTCCTGGTGGGGAGGTGGAAAG 2352
                ************************* **********************************

sad1-a          TGGATGCTTTTACGGGGCTTTCCGGGGAAGGGAACAAAGCTCAGGATTTTGTCTGCGGGC 2408
sad2-a          TGGATGCTTTTACGGGACTTTCCGGGGAAGGGAACAAAGCTCAGGAGTTTGTCTGTGGGC 2412
                **************** ***************************** ******** ****

sad1-a          TTCCTGCGAGGATTCGAAAGTTGGAGGAGAGGGCTGCGGGGAGGGCAAAGCAAACGTCGA 2468
sad2-a          TTCCAGCGAGGATTCGAAAATTGGAGGAGAGGGCTGCGGGGAGGGCAAAGCAAACGTCGA 2472
                **** ************** ****************************************

sad1-a          AATCTGTCCCGTTCAGCTGGATCTTCAGCAGAGAATTGGTACTCTAA 2515
sad2-a          AATCTGTCCCATTCAGCTGGATCTTCAGCAGAGAATTGGTACTCTAA 2519
                ********** ************************************
```

b

```
SAD1-A          MALKLNPVTTFPSTRSLNNFSSRSPRTFLMAASTFNSTSTKEAEKLKKSHGPPKEVHMQV 60
SAD2-A          MALKLNPVTTFPSTRSLNNFSSRSPRTFLMAASTFNSTSTKEAEKLKKSHGPPKEVHMQV 60
                ************************************************************

SAD1-A          THSMPPQKLEIFKSLEGWAEDVLLPHLKPVEKCWQPQDFLPEPESDGFEEQVKELRARAK 120
SAD2-A          THSMPPQKLEIFKSLEGWAEDVLLPHLKPVEKCWQPQDFLPEPESDGFEEQVKELRARAK 120
                ************************************************************

SAD1-A          ELPDDYFVVLVGDMITEEALPTYQTMLNTLDGVRDETGASLTPWAIWTRAWTAEENRHGD 180
SAD2-A          ELPDDYFVVLVGDMITEEALPTYQTMLNTLDGVRDETGASLTPWAIWTRAWTAEENRHGD 180
                ************************************************************

SAD1-A          LLNKYLYLSGRVDMRQIEKTIQYLIGSGMDPKTENNPYLGFIYTSFQERATFISHGNTAR 240
SAD2-A          LLNKYLYLSGRVDMRQIEKTIQYLIGSGMDPKTENNPYLGFIYTSFQERATFISHGNTAR 240
                ************************************************************

SAD1-A          LAKDHGDMKLAQICGIIAADEKRHETAYTKIVEKLFEIDPDGTVLALADMMRKKISMPAH 300
SAD2-A          LAKDHGDMKLAQICGIIAADEKRHETAYTKIVEKLFEIDPDGTVLALADMMRKKISMPAH 300
                ************************************************************

SAD1-A          LMYDGEDDNLFDNYSSVAQRIGVYTAKDYADILEFLVGRWKVDAFTGLSGEGNKAQDFVC 360
SAD2-A          LMYDGEDDNLFDNYSSVAQRIGVYTAKDYADILEFLVGRWKVDAFTGLSGEGNKAQEFVC 360
                ********************************************************:***

SAD1-A          GLPARIRKLEERAAGRAKQTSKSVPFSWIFSRELVL 396
SAD2-A          GLPARIRKLEERAAGRAKQTSKSVPFSWIFSRELVL 396
                ************************************
```

Fig S1. CLUSTAL alignment of (a) DNA sequences and (b) deduced amino acid sequences of *sad1*-a and *sad2*-a. Identical residues indicated by asterisks (*) and gaps are identified by dashes. Conserved amino acid substitutions are denoted with colon (:) and semi-conserved substitutions are indicated by a dot (.). Numbers on the right indicate the position number.
