# Supplementary material for: Genetic variation of six desaturase genes in flax and their impact on fatty acid composition
Source: Theor Appl Genet. 2013 Aug 9;126(10):2627–41. doi: 10.1007/s00122-013-2161-2 (PMC3782649; doi:10.1007/s00122-013-2161-2)
Supplement: Supplementary file 5 — Supplementary material 5 (PDF 146 kb) [file 122_2013_2161_MOESM5_ESM.pdf]

a

```
fad2a-a         ATGGGTGCCGGTGGCAGAATGTCAGTGCCTCCATCA------------TCCAAACCTATG 48
fad2b-a         ATGGGTGCTGGCGGAAGAATGGCCGTGCCTCCATCGAACAAGGCGGACTCCGAAACCTTT 60
                ******** ** ** ****** * ***********             *** ** *  *

fad2a-a         AAGAGGTCTCCTTACTCAAAGCCACCATTCACGCTCGGTGAGCTCAAGAAGGCCATTCCT 108
fad2b-a         AAGCGGTCTCCTTACTCAAAACCTCCCTTCACTCTTGGTGAGATCAAGAAAGCCGTCCCT 120
                *** **************** ** ** ***** ** ****** ******* *** * ***

fad2a-a         CCACACTGTTTCAAACGTTCAATCCCCCGATCGTTCGCCTACGTGGCGTACGACCTCACC 168
fad2b-a         CCACACTGCTTCAAAAGGTCCATCCCCCGCTCGTTCTCCTACGTGGCTTATGACCTCACC 180
                ******** ****** * ** ******** ****** ********** ** *********

fad2a-a         ATTGCAGCAATCTTCTACTACATCGCCACCACTTACTTCCACCTCCTCCCTAGCCCTCTC 228
fad2b-a         ATAGCCGCCATCTTCTACTACATCGCCACCACTTACATCCACCTCCTCCCCAATCCTCTC 240
                ** ** ** *************************** ************* *  ******

fad2a-a         AACTACCTCGCCTGGCCGGTCTACTGGGCCTGCCAGGGCTGCATCCTCACTGGAGTATGG 288
fad2b-a         TCCTACGTGGCGTGGCCGATCTACTGGGCCTGCCAAGGCTGCGTCCTCACTGGTGTCTGG 300
                  **** * ** ****** **************** ****** ********** ** ***

fad2a-a         GTGTTGGCTCACGAATGCGGTCACCATGCCTTCAGCGACTACCAGTGGCTCGACGACATG 348
fad2b-a         GTCCTAGCCCACGAATGCGGTCACCATGCCTTCAGCGACTACCAATGGCTCGACGACTTG 360
                **  * ** *********************************** ************ **

fad2a-a         GTTGGCTTCGTCCTCCATTCGTCCCTCCTTGTTCCTTACTTCTCCTGGAAGCACAGCCAC 408
fad2b-a         GTCGGCTTTGTCCTCCACTCATGCCTCATGGTACCCTACTTCTCGTGGAAGCACAGCCAC 420
                ** ***** ******** ** * **** * ** ** ******** ***************

fad2a-a         CGCCGCCACCATTCCAACACGGGATCGCTTGATCGTGATGAGGTGTTTGTCCCCAAGCAG 468
fad2b-a         CGTCGCCACCACTCCAATACTGGGTCCCTCGAACGAGACGAGGTTTTTGTCCCCAAGCAG 480
                ** ******** ***** ** ** ** ** ** ** ** ***** ***************

fad2a-a         AAGGCCGAAATCGGGTGGTACTCCAAGTACCTTAACAACCCACCTGGCCGTGTGATCACA 528
fad2b-a         AAATCAGCCATTGGCTGGCACTCAAAGTACCTCAACAACCCACCTGGCCGTGTGCTCACA 540
                **  * *  ** ** *** **** ******** ********************* *****

fad2a-a         TTGGCCGTCACATTAACGCTCGGTTGGCCTCTGTACTTGGCATTCAACGTCTCCGGGAGA 588
fad2b-a         CTTGCAGTCACTCTCACTCTCGGCTGGCCTTTGTACTTGGCATTCAACGTCTCTGGAAGG 600
                 * ** *****  * ** ***** ****** ********************** ** **

fad2a-a         CCATATGACCGGTTCGCATGCCATTTTGACCCTCACGGTCCGATTTACAATGATCGCGAG 648
fad2b-a         CCGTACGACCGGTTCGCCTGCCATTACGATCCTAAATCCCCCATCTACAACGACCGCGAG 660
                ** ** *********** *******  ** *** *    ** ** ***** ** ******

fad2a-a         CGTATGGAGATATACCTATCCGACGCAGGGATATTCACCGTGTGCTACATCCTATACAGA 708
fad2b-a         CGAACGGAGATATTCTTCTCCGATGCTGGCATCCTTGCTGTGAGCTTTGCGCTCTACAAG 720
                ** * ******** * * ***** ** ** **  *  * *** ***     ** ****

fad2a-a         CTCGTCCTCACGAAAGGACTCGTTTGGGTCGTGTCCATATACGGAGTCCCACTATTGATA 768
fad2b-a         CTTGCTGTCGCCAAGGGACTGGCTTGGGTGGTTTGTGTCTACGGAGTTCCACTCCTTGTA 780
                ** *   ** * ** ***** * ****** ** *   * ******** *****  *  **

fad2a-a         GTGAATGGATTCCTAGTCCTCATCACTTTCTTGCAGCACACGCATCCTTCTCTTCCGCAC 828
fad2b-a         GTGAATGGATTCCTTGTCTTGATCACTTTCTTGCAGCACACCCACCCATCATTGCCGCAC 840
                ************** *** * ******************** ** ** **  * ******

fad2a-a         TACAAGTCCTCCGAATGGGACTGGATGCGAGGCGCCCTCTCGACCGTGGATCGAGACTAC 888
fad2b-a         TACAAATCCTCCGAATGGGACTGGCTGAGAGGTGCTCTGGCGACCATGGACAGAGACTAC 900
                ***** ****************** ** **** ** **  ***** ****  ********

fad2a-a         GGGTTACTCAACACCGTGTTCCACAACATCACCGACACACATGTCGCGCACCATCTCTTC 948
fad2b-a         GGGTTTCTGAACACGGTGTTCCATAACATCACGGATACCCACGTGGCGCACCACCTGTTC 960
                ***** ** ***** ******** ******** ** ** ** ** ******** ** ***

fad2a-a         TCCACGATGCCTCATTACCACGCGATGGAGGCTACCAAGGCGATCAAGCCGGTTCTCGGG 1008
fad2b-a         TCGACGATGCCTCATTACCATGCAATGGAAGCTACAAAGGCGATCAAGCCGGTATTGGGA 1020
                ** ***************** ** ***** ***** *****************  * **
```

```
fad2a-a         GAGTATTACCAGTTCGATGGGACTCCCTTTGTGAAGGCCATGTGGAGGGAGGCAAAGGAG 1068
fad2b-a         GAGTACTACCAATTCGACGGGACTCCATTCATCAAGGCGATGTGGAGGGAGGCTAAGGAG 1080
                ***** ***** ***** ******** **  * ***** ************** ******

fad2a-a         TGCATCTATGTCGAGCCGGATGAAGGCGACCCCAGCCAAGGCGTGTTCTGGTACAACAAC 1128
fad2b-a         TGTGTTTATGTCGAGCCCGACGAAGGTGACCAGAACAAAGGCGTGTTCTGGTACAACAAC 1140
                **  * *********** ** ***** ****  * * ***********************

fad2a-a         AAGCTGTGA 1137
fad2b-a         AAGCTGTGA 1149
                *********
```

b

```
FAD2A-A         MGAGGRMSVPPS----SKPMKRSPYSKPPFTLGELKKAIPPHCFKRSIPRSFAYVAYDLT 56
FAD2B-A         MGAGGRMAVPPSNKADSETFKRSPYSKPPFTLGEIKKAVPPHCFKRSIPRSFSYVAYDLT 60
                *******:****    *:.:**************:***:*************:*******

FAD2A-A         IAAIFYYIATTYFHLLPSPLNYLAWPVYWACQGCILTGVWVLAHECGHHAFSDYQWLDDM 116
FAD2B-A         IAAIFYYIATTYIHLLPNPLSYVAWPIYWACQGCVLTGVWVLAHECGHHAFSDYQWLDDL 120
                ************:****.**.*:***:*******:************************:

FAD2A-A         VGFVLHSSLLVPYFSWKHSHRRHHSNTGSLDRDEVFVPKQKAEIGWYSKYLNNPPGRVIT 176
FAD2B-A         VGFVLHSCLMVPYFSWKHSHRRHHSNTGSLERDEVFVPKQKSAIGWHSKYLNNPPGRVLT 180
                *******.*:********************:**********: ***:***********:*

FAD2A-A         LAVTLTLGWPLYLAFNVSGRPYDRFACHFDPHGPIYNDRERMEIYLSDAGIFTVCYILYR 236
FAD2B-A         LAVTLTLGWPLYLAFNVSGRPYDRFACHYDPKSPIYNDRERTEIFFSDAGILAVSFALYK 240
                ****************************:**:.******** **::*****::*.: **:

FAD2A-A         LVLTKGLVWVVSIYGVPLLIVNGFLVLITFLQHTHPSLPHYKSSEWDWMRGALSTVDRDY 296
FAD2B-A         LAVAKGLAWVVCVYGVPLLVVNGFLVLITFLQHTHPSLPHYKSSEWDWLRGALATMDRDY 300
                *.::***.***.:******:****************************:****:*:****

FAD2A-A         GLLNTVFHNITDTHVAHHLFSTMPHYHAMEATKAIKPVLGEYYQFDGTPFVKAMWREAKE 356
FAD2B-A         GFLNTVFHNITDTHVAHHLFSTMPHYHAMEATKAIKPVLGEYYQFDGTPFIKAMWREAKE 360
                *:************************************************:*********

FAD2A-A         CIYVEPDEGDPSQGVFWYNNKL 378
FAD2B-A         CVYVEPDEGDQNKGVFWYNNKL 382
                *:******** .:*********
```

Fig S2. CLUSTAL alignment of (a) DNA sequences and (b) deduced amino acid sequences of *fad2a-a* and *fad2b-a*. Identical residues indicated by asterisks (*) and gaps are identified by dashes. Conserved amino acid substitutions are denoted with colon (:) and semi-conserved substitutions are indicated by a dot (.). Numbers on the right indicate the position number.
