# Supplementary material for: Genetic variation of six desaturase genes in flax and their impact on fatty acid composition
Source: Theor Appl Genet. 2013 Aug 9;126(10):2627–41. doi: 10.1007/s00122-013-2161-2 (PMC3782649; doi:10.1007/s00122-013-2161-2)
Supplement: Supplementary file 6 — Supplementary material 6 (PDF 156 kb) [file 122_2013_2161_MOESM6_ESM.pdf]

a

```
fad3a-a          ATGAGCCCTCCAAACTCAATGAGTCCCGCCACCAACGGCAGCACCAATGGTGTGGCTATC 60
fad3b-a          ATGAGCCCTCCAAACTCAATGAGTCCCACCACCAACGGCA------ATGGTGTGGCTATG 54
                 *************************** ************      *************

fad3a-a          AATGGGGCGAAGAAGCTACTCGATTTCGACCCGAGTGCTGCTCCCCCTTTCAAGATTGCA 120
fad3b-a          AATGGGGCGAAGAAGCAGCTCGATTTCGACCCGAGTGCTGCCCCCCCTTTCAAGATTGCA 114
                 ****************  *********************** ******************

fad3a-a          GACATCCGTGCTGCAATCCCGCCGCATTGTTGGGTGAAGAACCCCTGGAGGTCACTCAGC 180
fad3b-a          GACATCCGTGCTGCAATTCCGCCGCATTGCTGGGTGAAGAACCCCTGGAGGTCGCTCAGC 174
                 ***************** *********** *********************** ******

fad3a-a          TACGTCCTGAGAGACCTCCTGGTCATCCTCAGCTTCGCCGTTGCGGCGACAAAGCTGGAC 240
fad3b-a          TACGTCCTGAGAGACCTCCTTGTCATCCTCAGCTTCGCCGTTGCGGCGGCAAAGCTGGAC 234
                 ******************** *************************** ***********

fad3a-a          AGCTGGACTGTCTGGCCTCTCTACTGGATTGCTCAAGGAACCATGTTCTGGGCAGTCTTT 300
fad3b-a          AGCTGGACTTTCTGGCCTCTTTACTGGGTTGCTCAAGGAACCATGTTCTGGGCAGTCTTT 294
                 ********* ********** ****** ********************************

fad3a-a          GTTCTTGGACATGATTGGTAA-----TTTCACATGATCTTTCTGGTAATGTGGGTTTTCT 355
fad3b-a          GTTCTTGGACATGATTGGTAAACTAATTTCACATTTTCTTTCTGGTAATGTGGGTTTT-- 352
                 *********************     ********  **********************

fad3a-a          TTTCTTATTGAAAAAGATTAAAACTTTTTATCTGGGCTGTTGCATGCAGTGGCCATGGGA 415
fad3b-a          ------ATTGAAAAAGATTAAAACTTTTTATCTGGGTTGTTGCATGCAGTGGCCATGGGA 406
                       ****************************** ***********************

fad3a-a          GCTTCTCAGACAGTTGGTTGTTGAACAACGTGATGGGACATATACTCCATTCCTCAATCC 475
fad3b-a          GCTTCTCAGACATCTGGTTGTTGAACAATGTGATGGGACATATACTCCATTCCTCAATCC 466
                 ************  ************** *******************************

fad3a-a          TCGTACCTTACCATGGATGGTATTGTAACTATTGTTCGATATTCGATTATGATTACTGTT 535
fad3b-a          TCGTACCTTACCATGGATGGTATTGTAACTATTGTTCAATATTAGATT---ATTGCTAGT 523
                 ************************************* ***** ****   *** **  *

fad3a-a          CTTTCAGATGAAGAATCTGTACCCTAATTGTTTTTTGTT--------ACCAGGAGAATTA 587
fad3b-a          TCTTCAGCTGAAGAATCCAAACCCTAATTTTCTTTTTCTGAATATTGACCAGGAGAATTA 583
                   ***** *********   ********* * ****  *        *************

fad3a-a          GCCACAAGACCCATCACCAGAATCACGGCAATGTGGAGAAAGATGAATCCTGGGTTCCAG 647
fad3b-a          GCCACAAGACCCATCACCAGAATCACGGCAATGTGGAGAAAGATGAATCCTGGGTTCCTG 643
                 ********************************************************** *

fad3a-a          TAAGTTGACATGCAGTTTGCTCTAAAA-TGCAGAGTCCTCTGTTTTTTGTGTGTTCTTGT 706
fad3b-a          TAAGTTGACATGCAGTTTGCTGTAAAAATGCAGAGTGCTCTGTTTTTT-TGTGTTCTTGT 702
                 ********************* ***** ******** *********** ***********

fad3a-a          GCTTTAATGACGATGATAATGAAATTG---AAATTTGTAATAGCTGCCGGAGAAGGTGTA 763
fad3b-a          GCTTTAATGGTGATAATAATGAAATTGTTGAAA--TGTAACAGCTACCGGAGAAAGTGTA 760
                 *********  *** ************   ***  ***** **** ******** *****

fad3a-a          CAAGAGCTTGGATACCGGCACCAAGTTCATGAGGTTCACCATCCCTCTCCCAATGTTTGC 823
fad3b-a          CAAGAGCTTGGATACCAGCACTAAGTTCATGAGGTTCACCATTCCTCTCCCAATGTTTGC 820
                 **************** **** ******************** *****************

fad3a-a          GTATCCTATCTACTTGGTAAGTAAACAGACTGA-----CTCCAAAGTAGGAACTAATGAC 878
fad3b-a          TTATCCTATCTACTTGGTAAGTAAAGAGACTGATAAGACTCCAAAGTAGGAATTAATGAC 880
                  ************************ *******     ************** *******

fad3a-a          AATTTTGGACCCGACCTGGTTTGGTTGACTCGGGTCGATATGTTTCGGGTGGGTAATTAC 938
fad3b-a          AATTTTGGACCCGA----GCTT--CCGACTCGGGTCGATTTATTTCGGGTGGGTACTTAC 934
                 **************    * **    ************* * ************* ****

fad3a-a          CCGATCTGGCGATGGGTGTGCGGCGGACATTGTCTTGCTCGTGGTCCACCCCGCTCCCAA 998
fad3b-a          CCGAT--------------GCGGCGGACATTGTTTTGCTCGTGGTCCACCTCGCTCCCAA 980
                 *****              ************** **************** *********
```

```
fad3a-a        CCCGCCCCATTCTTGACGAAAAAGATTTCGGAATATGTATCAACAGAAAAATCTAGTTTT 1058
fad3b-a        CCCGCCCCATTCTTGACGAAAAAGATTACGGAATATGTATCAACAGAAATATCTAGTTTT 1040
               *************************** ********************* **********

fad3a-a        TATGTTACTAGTTTTCTGTATTTCCATGTTTTTTCC-TCAATTCTAGCCGGAATTTGAAT 1117
fad3b-a        TATGTTACTAGTTTTCTGTATTTCCGTGTTTTTCCCCTCAATTCCGTCAGAAATTTGAAT 1100
               ************************* ******* ** *******   * * *********

fad3a-a        TCAAACTGAAATCGGGTAATTCCGTCCATAACAAAACGGAATTGGGCAGCCGTAATTAGT 1177
fad3b-a        TCAAACTGACATTGGGTAATTCTGTCCATAAGAGAACGGAATTGGGAAGCCGTAATTAGT 1160
               ********* ** ********* ******** * ************ *************

fad3a-a        TGAAACTAGACCTCAATTTTGGCGGAATTGGACCCGGCCATTTTTTACGTTTGCAAAACG 1237
fad3b-a        TGGAATTAGACCTCGATTTCGGCGGAATTGGACCCGGCCATTTTTCGCGTCCG------- 1213
               ** ** ******** **** *************************  ***  *

fad3a-a        GAAAACGTTTTTCTTTTGTAAAGCGCAAAATGAAAAACGTATCTAGTGGAATTATTGGAC 1297
fad3b-a        -------------------------------GAAAAGCGTTTCCAGTGGAGTTAGACG-C 1241
                                              ***** *** ** ****** ***   * *

fad3a-a        CCATCTAGAATGGGTCCAATTCCACCCCAATTTCGGCTCCAATTCATGCCCGGAAAACAC 1357
fad3b-a        CCATCTAGAATGGGTCCAATTCCACCCCAATTTCGGGGCTACCT-----------ATTTT 1290
               ************************************  * *  *           *

fad3a-a        TACTGT-CATGCATTTTA-ATCTTGTATGGTTTTACCCCAATGGATGCAGCGATGGATCC 1415
fad3b-a        TAGTGTACATGCATTTTAGATCTTTTACGGTCTTACCCC--------------------- 1329
               ** *** *********** ***** ** *** *******

fad3a-a        GGACGATTTTTAAAATATTATCGGGTTAAATTTAAAAATATCTTAAAACTATAAGAAAAA 1475
fad3b-a        ------------------------------------------------------------

fad3a-a        AATAACCAATTTTAAAGAATAAAAGAACTGGACACATATGACGGGTGTCGTGGATGGATG 1535
fad3b-a        ----------------------------------------------------GATGGATA 1337
                                                                   *******

fad3a-a        TACTTGTCCCGCTCTATTAAAGGCTGATAATATACAGGTCAACGGTGAATGAAGGTTAGA 1595
fad3b-a        TTCTTGTCGCGCTCTATTAAAAACTGACAATATACAGGTTGATTGTGAATGAAGATTAGG 1397
               * ****** ************  **** ***********  *  ********** ****

fad3a-a        TGCGCTATTGGATTTGAATCCGATATGAAATGATAATTTTGGACACGATCTGTTTTGGGT 1655
fad3b-a        TGC-----TG---------------------------------CACGCTCT--------- 1410
               ***     **                                 **** ***

fad3a-a        GGGTAATATTTGATCTAGGGATGGCTCGTGCTCCAAACCGCACCAAAACCGCCTAATTCT 1715
fad3b-a        -------------------------TCGTGTTCCAACCCGCACCCAAACCGCCCCATTCT 1445
                                        ***** ***** ******* ********  *****

fad3a-a        CGACCAAAAAGATTTTATGAATACATATCAACAGAAAAATCTAGTTTTCATGTTACTAGT 1775
fad3b-a        CGACCGAAAAGATTTTATGAATACATATCTACAGAAAAATCTAGTTGTCATGTCACTAGT 1505
               ***** *********************** **************** ****** ******

fad3a-a        TTTATGTACAACAATATTAGGTGTCGTTTTCCCAGCCTTTTTCTTCAATTCCGGCCGGAA 1835
fad3b-a        TTAATGTACAACAGTAT-AGGTGTCGTTTTCCGCGTCTTTTTCTTCAATTCCGGCTGGAA 1564
               ** ********** *** **************  * ******************* ****

fad3a-a        TTCGCATTCAAACCGGAATTGGATGGAATCGGTATACCTCGTCACGGATGCATTGTCAAT 1895
fad3b-a        TTCGCATTCAAACAGGAATTGGATGGAATTGGT---------CCCGGATGCATAGTCATT 1615
               ************* *************** ***         * ********* **** *

fad3a-a        TCCTAGTTAGTTTCATGGTTTTGAAACCAATCAATCTATTCTATATGGTTTTGATTAACA 1955
fad3b-a        TCCCAGGCAGTTTCATGGTTTTATAACCAATCAATCTAATCT-TATGCTTTTGATAAACA 1674
               *** **  **************  ************** *** **** ******* ****

fad3a-a        GTGGAGGAGAAGTCCGGGGAAGAAAGGGTCGCATTTCAACCCATACAGTGACCTGTTCGC 2015
fad3b-a        GTGGACGAGAAGTCCGGGGAAGAAAGGGTCGCATTTCAACCCATACAGCGACCTATTCGC 1734
               ***** ****************************************** ***** *****

fad3a-a        ACCGAACGAGAGGACATCGGTCATGATTTCGACATTGTGCTGGACAGCCATGGCCTTACT 2075
fad3b-a        ACCAAACGAGAGGGCAGCGGTCTTGATTTCAACATTGTGCTGGACAGCCATGGCCTTACT 1794
               *** ********* ** ***** ******* *****************************
```

```
fad3a-a        CCTCTGCTACTCATCGTTCATCTACGGCTTCCTTCCGGTCTTCAAAATCTACGGCGTCCC 2135
fad3b-a        CCTCTGCTACTCATCGTTCATATACGGCTTCGCTCCGGTCCTCAAAATCTACGGCGTACC 1854
               ********************* *********  ******* **************** **

fad3a-a        TTATCTAATATTCGTGGCGTGGCTCGACATGGTGACCTACCTTCACCACCACGGGTACGA 2195
fad3b-a        TTATCTGATATTCGTGGCATGGCTCGACATGGTGACCTACCTTCATCACCACGGGTACGA 1914
               ****** *********** ************************** **************

fad3a-a        GCAGAAGCTGCCGTGGTACAGAGGCAAAGAGTGGAGCTACCTACGTGGAGGGCTGACGAC 2255
fad3b-a        GCAGAAGCTGCCGTGGTACAGAGGCAAAGAATGGAGCTACCTACGTGGAGGGCTGACGAC 1974
               ****************************** *****************************

fad3a-a        CGTCGATCGAGATTACGGGGTCATCAACAACATCCACCATGACATTGGCACCCATGTTAT 2315
fad3b-a        CGTTGATCGAGATTACGGGGTCATCAACAACATCCACCATGACATTGGCACCCATGTCAT 2034
               *** ***************************************************** **

fad3a-a        TCACCATCTCTTCCCTCAAATGCCACACTATCACCTAGTCGAAGCGGTAAGGAGGTCTTG 2375
fad3b-a        TCACCATCTCTTCCCTCAAATGCCACACTATCACCTTGTGGAAGCGGTAAACAA--TTTG 2092
               ************************************ ** **********  *    ***

fad3a-a        ATTATTAACTTAATGTTTTTGTTGTTATAATTTGAGTCCGATTCTGGAGTCAGGGGATTT 2435
fad3b-a        ATTATTAATTTACTGTTTTTGTTGTTATAATTTGAGTC-------------GGGAGATTT 2139
               ******** *** *************************              ** *****

fad3a-a        CCTTCTTGGATCCGATCCAGGATCAAGCTGGTCCCTTGAATTTCTATATGATCT------ 2489
fad3b-a        CCTTCCTAAATCCGATCCCTGGTCAATCTTGGCCCTTGAATCTTCATATAATCTAAAAAT 2199
               ***** *  *********  * **** ** * ********* *  **** ****

fad3a-a        -TATATTAATTAAGGATAATGTGGTCATATGTTTTTAAATATTTTTGTT----------- 2537
fad3b-a        CTAGATTAATCAGGAACAATATGATCATGTTGTTTAAACTAATTTTGTTGGACCATAACC 2259
                ** ****** * * * *** ** **** *  *** ** ** *******

fad3a-a        TACC-------------------------------------ATCATTTTCGATCACCGGA 2560
fad3b-a        TACCGCCAACTGATGGACCACCGTCTCTGGTTACCGGACCCATCATTTCCGGTTACCAAG 2319
               ****                                     ******* ** * ***

fad3a-a        AAATGTCCTGAGCAGTTTTCCGGTCACTTTAACCTCCATTGAC--AAATTTTTTCACCCA 2618
fad3b-a        AAGTTTCTCGATCAGTTTTCCGGTTACTTTGACCTGCGTTGAGGAAAATTCTTTCACCCA 2379
               ** * **  ** ************ ***** **** * ****   ***** *********

fad3a-a        CATGATCACCCTAGCCGGGTTTACGTTTATTGAAAATTTTTATTTTTTTGAATTTTTTTT 2678
fad3b-a        CGTAAACACTGTCGTCAACTTTACGTTTCTGGAAAGTTTTT------------------C 2421
               * * * ***  * * *   ********* * **** *****

fad3a-a        CGATGACCAACTGTACAACTTTGTATTGAAAGTTGTATGGATCATACAAATGTGTATG-- 2736
fad3b-a        CGATGATTGGCCGTACAATTTTGTACGAAGAGTTGTACGGATCATATAAATGTGTATAAG 2481
               ******    * ****** ******   * ******* ******** **********

fad3a-a        --TACAAAAGTATATTCT-AAGTACTATACTAAGCATTACTTA-GTATTACGTTTC-TAC 2791
fad3b-a        TTTCTAGAAATCCGTACTGAAATA-TATAC-ATATTTGACTTTTGTATAAAGTGTAATAC 2539
                 *  * ** *   * ** ** ** ***** *    * ****  **** * ** *  ***

fad3a-a        -AAAC-CTATAGAGAAATGCATACAATTTTGTATAGAACTTAGTATACACGT------AG 2843
fad3b-a        TAAATACTATACT-AAGTGC-TGTA-CTCAGTATGATACTTAGTACACACATTTGTATGA 2596
                ***  *****   ** *** *  *  *  ****   ******** **** *

fad3a-a        CTGTGAAATGTCAATTTC-CCTCCGTATTTTCAG-----AGAC--AAGACATGATTTTTA 2895
fad3b-a        CTATGAAATGTCAATTTTGCCCTTATATTCTCAGCCGTTAGATCTAAGACACAGTTTTTA 2656
               ** **************  **    **** ****     ***   ******   ******

fad3a-a        GACTGGC--AGATTTTT------TTT----ATCGGAT-----AG--ATTTCTCCAACTTC 2936
fad3b-a        TAC-GGCTGAAATTTGTGGGGGCTTTGTAGATCGGATCCATAAGTCATTTCTT-GGCTCA 2714
                ** ***  * **** *      ***    *******     **  ******    **

fad3a-a        AGATTCGGACTGGATTATTAACTATATTATTCATCAACTCTGACGTTTGATGTTGCATGT 2996
fad3b-a        AGATTCGGACTCGATTATTAACTATATTATTCATCAACTCTGACGTTTGATGTTGCATGT 2774
               *********** ************************************************

fad3a-a        GACAGACTCAGGCAGCGAAGCACGTGCTGGGGAAGTACTACAGAGAACCGAAGAAATCAG 3056
fad3b-a        GACAGACTCAGGCAGCGAAGCACGTGCTGGGGAAGTACTACAGAGAGCCGAAGAAATCAG 2834
```

```
                    ********************************************** *************

fad3a-a         GGCCTTTCCCATTCCACTTGTTTGGGTACTTGGTGAGGAGCCTGGGCGAGGATCACTACG 3116
fad3b-a         GGCCTTTCCCATTCCACTTGTTTGGGTACTTGGTAAGGAGCCTGGGCGAGGATCACTACG 2894
                ********************************** *************************

fad3a-a         TTAGCGATACAGGCGACGTCGTTTTCTATCAATCTGACCCACATATTCCCAAGTTCCCTA 3176
fad3b-a         TTAGCGACACAGGCGACGTCGTTTTCTATCAGTCTGACCCACATATTCCCAAGTTCCGTA 2954
                ******* *********************** ************************* **

fad3a-a         CCAG---TGCCACCACCAAGTCCAAATCTAGCTGA 3208
fad3b-a         CCAGCAGTGCCACCACCAAGTCCAAATCCAGCTGA 2989
                ****   ********************* ******
```

b

```
FAD3A-A         MSPPNSMSPATNGSTNGVAINGAKKLLDFDPSAAPPFKIADIRAAIPPHCWVKNPWRSLS 60
FAD3B-A         MSPPNSMSPTTNG--NGVAMNGAKKQLDFDPSAAPPFKIADIRAAIPPHCWVKNPWRSLS 58
                *********:***  ****:***** **********************************

FAD3A-A         YVLRDLLVILSFAVAATKLDSWTVWPLYWIAQGTMFWAVFVLGHDCGHGSFSDSWLLNNV 120
FAD3B-A         YVLRDLLVILSFAVAAAKLDSWTFWPLYWVAQGTMFWAVFVLGHDCGHGSFSDIWLLNNV 118
                ****************:******.*****:*********************** ******

FAD3A-A         MGHILHSSILVPYHGWRISHKTHHQNHGNVEKDESWVPLPEKVYKSLDTGTKFMRFTIPL 180
FAD3B-A         MGHILHSSILVPYHGWRISHKTHHQNHGNVEKDESWVPLPEKVYKSLDTSTKFMRFTIPL 178
                *************************************************.**********

FAD3A-A         PMFAYPIYLWRRSPGKKGSHFNPYSDLFAPNERTSVMISTLCWTAMALLLCYSSFIYGFL 240
FAD3B-A         PMFAYPIYLWTRSPGKKGSHFNPYSDLFAPNERAAVLISTLCWTAMALLLCYSSFIYGFA 238
                ********** **********************::*:**********************

FAD3A-A         PVFKIYGVPYLIFVAWLDMVTYLHHHGYEQKLPWYRGKEWSYLRGGLTTVDRDYGVINNI 300
FAD3B-A         PVLKIYGVPYLIFVAWLDMVTYLHHHGYEQKLPWYRGKEWSYLRGGLTTVDRDYGVINNI 298
                **:*********************************************************

FAD3A-A         HHDIGTHVIHHLFPQMPHYHLVEATQAAKHVLGKYYREPKKSGPFPFHLFGYLVRSLGED 360
FAD3B-A         HHDIGTHVIHHLFPQMPHYHLVEATQAAKHVLGKYYREPKKSGPFPFHLFGYLVRSLGED 358
                ************************************************************

FAD3A-A         HYVSDTGDVVFYQSDPHIPKFPTS-ATTKSKSS 392
FAD3B-A         HYVSDTGDVVFYQSDPHIPKFRTSSATTKSKSS 391
                ********************* ** ********
```

Fig S3. CLUSTAL alignment of (a) DNA sequences and (b) deduced amino acid sequences of *fad3a-a* and *fad3b-a*. Identical residues indicated by asterisks (*) and gaps are identified by dashes. Conserved amino acid substitutions are denoted with colon (:) and semi-conserved substitutions are indicated by a dot (.). Numbers on the right indicate the position number.
