# Supplementary material for: Genetic variation of six desaturase genes in flax and their impact on fatty acid composition
Source: Theor Appl Genet. 2013 Aug 9;126(10):2627–41. doi: 10.1007/s00122-013-2161-2 (PMC3782649; doi:10.1007/s00122-013-2161-2)
Supplement: Supplementary file 7 — Supplementary material 7 (PDF 91 kb) [file 122_2013_2161_MOESM7_ESM.pdf]

Table S4. Summary of SNPs and indels identified from *fad3a* allele 13, 14 and 15 (assembled into contig 2)

Accession	Exon I		Intron I		Exon II		Intron II		Exon III		Intron III		Exon IV		Intron IV		Exon V		Intron V		Exon VI		Allele	Isoform
	SNP	Indel	SNP	Indel	SNP	Indel	SNP	Indel	SNP	Indel	SNP	Indel	SNP	Indel	SNP	Indel	SNP	Indel	SNP	Indel	SNP	Indel		
CN96846	3	-	3	1	2	-	2	1	-	-	4	1	1	-	14	4	1	-	66	11	3	-	13	C
UGG102-2	3	-	3	1	2	-	2	1	-	-	4	1	1	-	15	4	1	-	65	11	3	-	14	C
CN97351	-	-	-	-	-	-	-	-	-	-	-	1	1	-	7	2	1	-	65	11	3	-	15	F
