# Supplementary material for: Genetic variation of six desaturase genes in flax and their impact on fatty acid composition
Source: Theor Appl Genet. 2013 Aug 9;126(10):2627–41. doi: 10.1007/s00122-013-2161-2 (PMC3782649; doi:10.1007/s00122-013-2161-2)
Supplement: Supplementary file 10 — Supplementary material 10 (PDF 26 kb) [file 122_2013_2161_MOESM10_ESM.pdf]

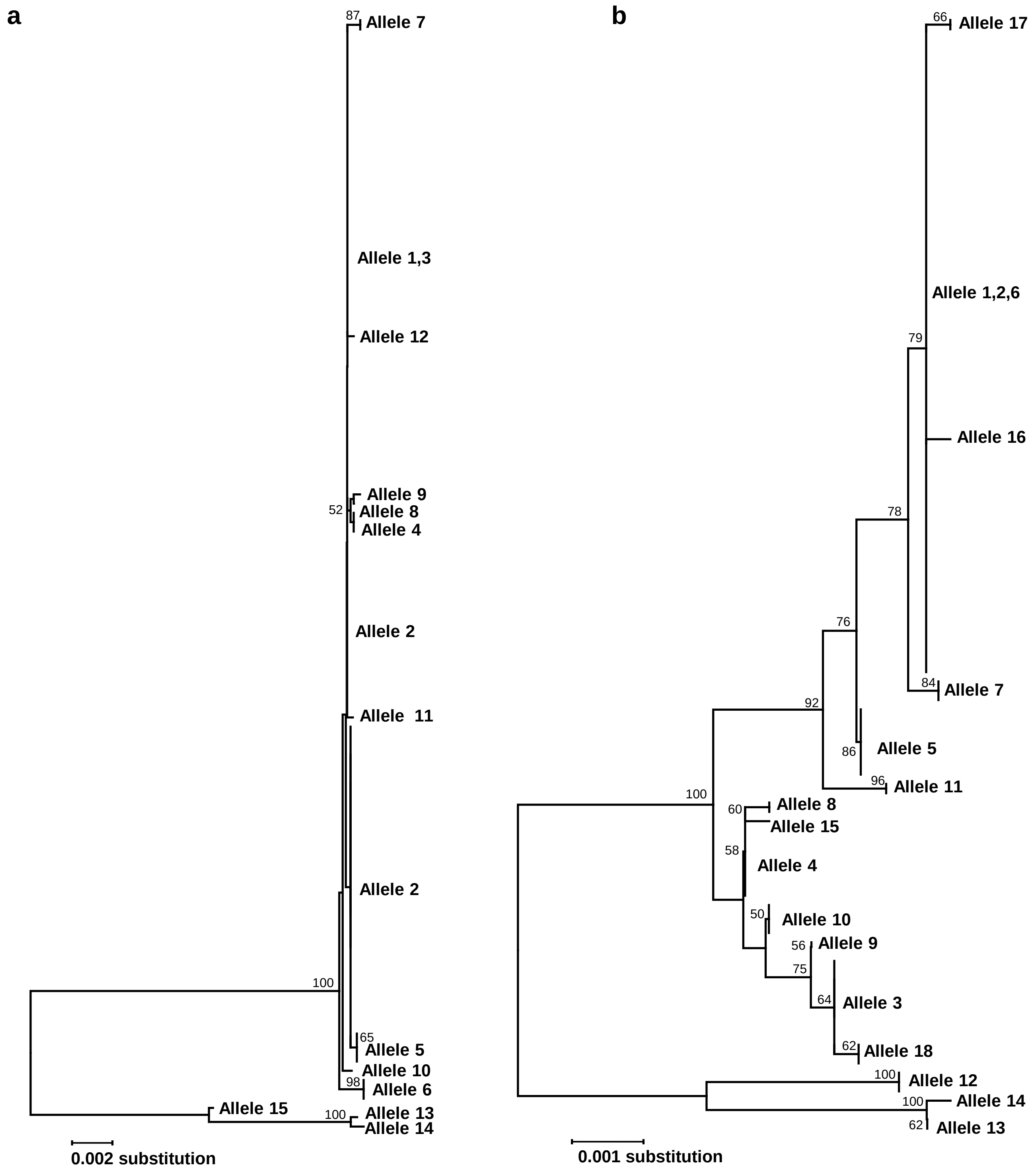

Fig S6. Neighbour-joining tree of (a) *fad3a* and (b) *fad3b* full length gene sequences from 120 accessions of flax. Accessions are identified by their allele number and vertical branch length represents number of accessions. Bootstrap values greater than 50 were shown.
