# Supplementary material for: Genetic variation of six desaturase genes in flax and their impact on fatty acid composition
Source: Theor Appl Genet. 2013 Aug 9;126(10):2627–41. doi: 10.1007/s00122-013-2161-2 (PMC3782649; doi:10.1007/s00122-013-2161-2)
Supplement: Supplementary file 11 — Supplementary material 11 (PDF 10 kb) [file 122_2013_2161_MOESM11_ESM.pdf]

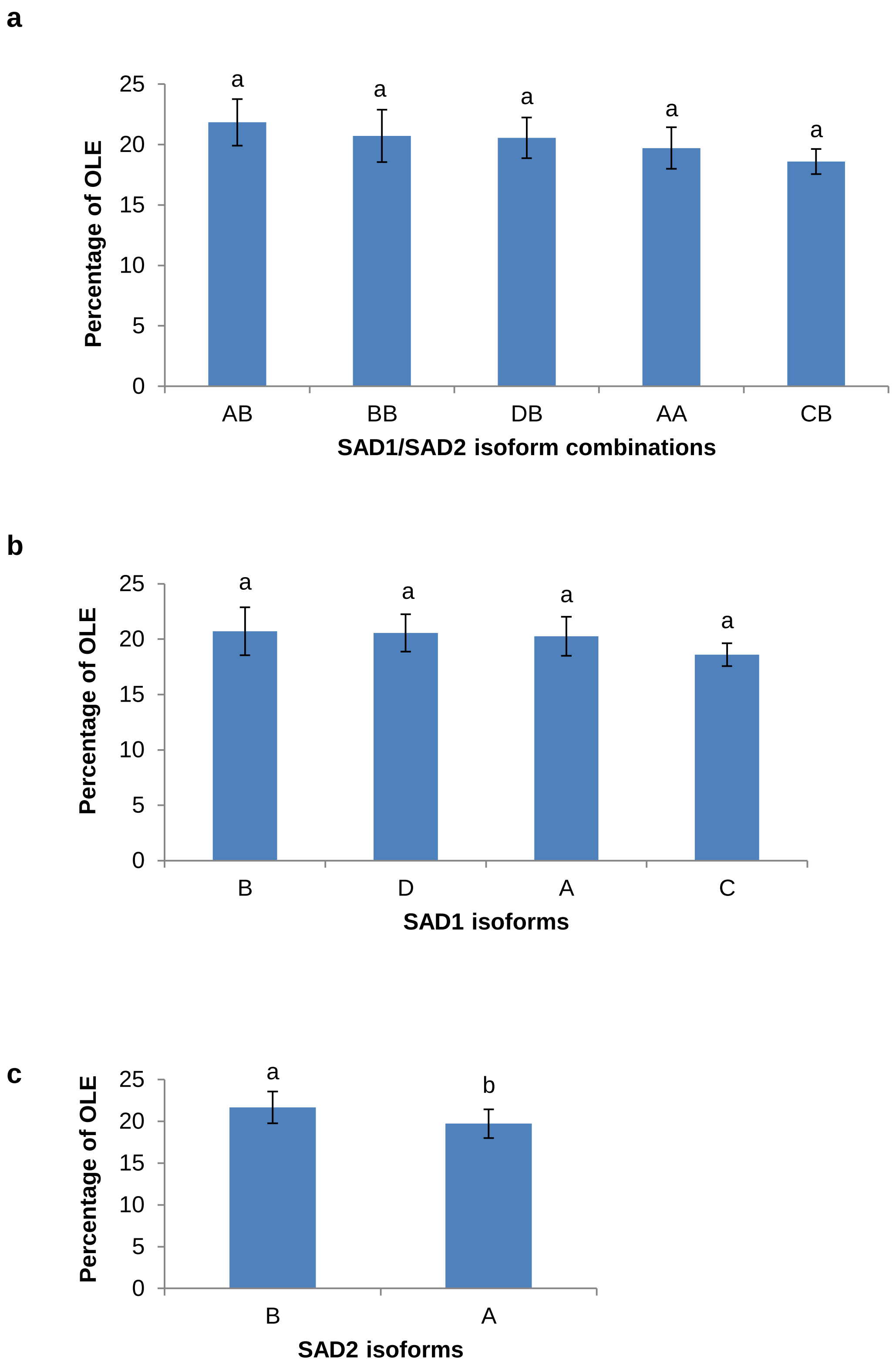

Fig S7. Association between OLE and the predicted isoforms of (a) SAD1/2 and OLE content, (b) SAD1 and OLE content and (c) SAD2. Vertical bars represent standard error of the mean. Letters on top of the bar indicate statistical significance of Duncan's multiple range tests.
