# Supplementary material for: Genetic variation of six desaturase genes in flax and their impact on fatty acid composition
Source: Theor Appl Genet. 2013 Aug 9;126(10):2627–41. doi: 10.1007/s00122-013-2161-2 (PMC3782649; doi:10.1007/s00122-013-2161-2)
Supplement: Supplementary file 12 — Supplementary material 12 (PDF 11 kb) [file 122_2013_2161_MOESM12_ESM.pdf]

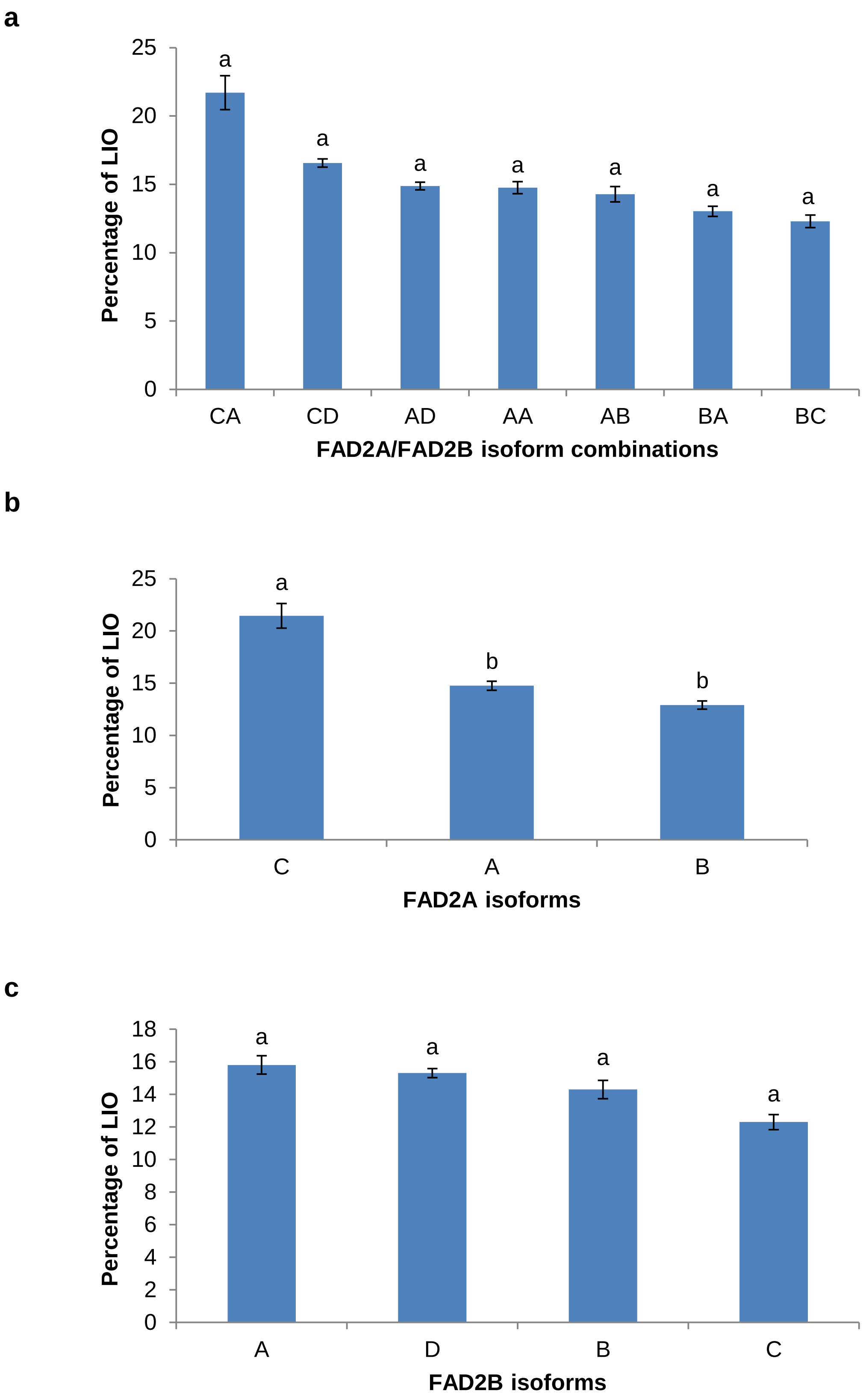

Fig S8. Association between LIO and the predicted isoforms of (a) FAD2A/B and LIO content, (b) FAD2A and LIO content and (c) FAD2B. Vertical bars represent standard error of the mean. Letters on top of the bar indicate statistical significance of Duncan's multiple range tests.
