# Supplementary material for: Genetic variation of six desaturase genes in flax and their impact on fatty acid composition
Source: Theor Appl Genet. 2013 Aug 9;126(10):2627–41. doi: 10.1007/s00122-013-2161-2 (PMC3782649; doi:10.1007/s00122-013-2161-2)
Supplement: Supplementary file 13 — Supplementary material 13 (PDF 96 kb) [file 122_2013_2161_MOESM13_ESM.pdf]

Table S5. Effect of SAD and FAD isoforms identified from the accessions representing the non-mutant flax germplasm on palmitic, stearic, oleic, linoleic and linolenic acid composition, oil content and iodine value

Trait	Genes/gene	*P*-value	Predicted isoforms or combinations
Palmitic acid (PAL)	*sad1*	0.5153	
	sad2	0.9991	
	fad2a	0.0169*	(5.51-5.95)[a]
	fad2b	0.8519	
	fad3a	0.8295	
	fad3b	0.0364*	[E(6.37)][a], [D,G(5.78-5.73)][ab], [A(5.47)][b]
	sad1/sad2	0.6638	
	fad2a/fad2b	0.1652	
	fad3a/fad3b	0.1961	
Stearic acid (STE)	*sad1*	0.1955	
	sad2	0.0955	
	fad2a	0.3710	
	fad2b	0.1749	
	fad3a	0.6480	
	fad3b	0.0701	
	sad1/sad2	0.0551	
	fad2a/fad2b	0.3845	
	fad3a/fad3b	0.1845	
Oleic acid (OLE)	*sad1*	0.9674	
	sad2	0.0118*	[B (21.77)][a], [A(19.87)][b]
	fad2a	0.0144*	[A(20.91)][a], [C(18.62)][ab], [B(17.90)][b]
	fad2b	0.2806	
	fad3a	0.5581	
	fad3b	0.0003*	(19.43-22.64)[a]
	sad1/sad2	0.1146	
	fad2a/fad2b	0.0097*	(15.59-21.12)[a]
	fad3a/fad3b	0.0021*	(19.23-24.18)[a]
Linoleic acid (LIO)	*sad1*	0.7191	
	sad2	0.7904	
	fad2a	0.1604	
	fad2b	0.4892	
	fad3a	0.6222	
	fad3b	<.0001*	[G(14.87)][a], [A,E(14.43-13.51)][ab], [D(12.33)][b]
	sad1/sad2	0.8547	
	fad2a/fad2b	0.4840	
	fad3a/fad3b	0.0004*	(11.76-14.87)[a]
Linolenic acid (LIN)	*sad1*	0.9777	
	sad2	0.0799	
	fad2a	0.0770	
	fad2b	0.4077	

	fad3a	0.6365	
	fad3b	0.0743	
	sad1/sad2	0.4339	
	fad2a/fad2b	0.0374*	(53.25-61.01)[a]
	fad3a/fad3b	0.1772	
Oil content (OIL)	*sad1*	0.9563	
	sad2	0.6414	
	fad2a	0.0004*	[C,A(44.07-42.68)][a], [B(39.89)][b]
	fad2b	0.2268	
	fad3a	0.1946	
	fad3b	0.0569	
	sad1/sad2	0.9804	
	fad2a/fad2b	0.0078*	[CA,CD (44.13-43.10)][a], [AA, AB, AD, BA(42.73-40.17)][b], [BC(38.52)][b]
	fad3a/fad3b	0.0599	
Iodine value (IOD)	*sad1*	0.9785	
	sad2	0.1757	
	fad2a	0.1898	
	fad2b	0.2593	
	fad3a	0.5835	
	fad3b	0.0016*	(183.70-189.88)[a]
	sad1/sad2	0.7473	
	fad2a/fad2b	0.0932	
	fad3a/fad3b	0.0081*	(179.11-190.68)[a]

[1]Means for the isoform(s) or isoform combinations are in bracket. They represent data collected from two locations during three years. Superscript letters indicate statistical significance of Duncan's multiple range tests. *Statistical significance ($p < 0.05$)
